# Synaptic mechanisms underlying onset and progression of memory deficits caused by hippocampal and midbrain synucleinopathy

**DOI:** 10.1038/s41531-023-00520-1

**Published:** 2023-06-16

**Authors:** Attilio Iemolo, Maria De Risi, Nadia Giordano, Giulia Torromino, Cristina Somma, Diletta Cavezza, Martina Colucci, Maria Mancini, Antonio de Iure, Rocco Granata, Barbara Picconi, Paolo Calabresi, Elvira De Leonibus

**Affiliations:** 1 Telethon Institute of Genetics and Medicine, Via dei Campi Flegrei 34, Pozzuoli, Naples Italy; 2 Institute of Genetics and Biophysics (IGB), Consiglio Nazionale delle Ricerche (CNR), via Pietro Castellino 111, Naples, Italy; 3 Institute of Biochemistry and Cell Biology, Consiglio Nazionale delle Ricerche (CNR), Via Ramarini 33, Monterotondo Scalo, Rome, Italy; 4 University of Naples Federico II, Department of Humanistic Studies, Naples, Italy; 5 Institute of Neuroscience (IN), Consiglio Nazionale delle Ricerche (CNR), via Raoul Follereau 3, Vedano al Lambro, Monza e Brianza Italy; 6 Lab. Experimental Neurophysiology, IRCCS San Raffaele, Rome, 00166 Italy; 7 Telematic University San Raffaele, Rome, 00166 Italy; 8 Neurological Clinic, Fondazione Policlinico Universitario Agostino Gemelli IRCCS, 00168 Rome, Italy; 9 Neurology, Department of Neuroscience, Faculty of Medicine, Università Cattolica del “Sacro Cuore”, 00168 Rome, Italy

**Keywords:** Parkinson's disease, Parkinson's disease

## Abstract

Cognitive deficits, including working memory, and visuospatial deficits are common and debilitating in Parkinson’s disease. α-synucleinopathy in the hippocampus and cortex is considered as the major risk factor. However, little is known about the progression and specific synaptic mechanisms underlying the memory deficits induced by α-synucleinopathy. Here, we tested the hypothesis that pathologic α-Synuclein (α-Syn), initiated in different brain regions, leads to distinct onset and progression of the pathology. We report that overexpression of human α-Syn in the murine mesencephalon leads to late onset memory impairment and sensorimotor deficits accompanied by reduced dopamine D1 expression in the hippocampus. In contrast, human α-Syn overexpression in the hippocampus leads to early memory impairment, altered synaptic transmission and plasticity, and decreased expression of GluA1 AMPA-type glutamate receptors. These findings identify the synaptic mechanisms leading to memory impairment induced by hippocampal α-synucleinopathy and provide functional evidence of the major neuronal networks involved in disease progression.

## Introduction

The abnormal and progressive neuronal deposition of the protein alpha-Synuclein (α-Syn), in the form of Lewy bodies and Lewy neurites, characterizes α-synucleinopathies that include Parkinson’s disease (PD), Parkinson’s disease dementia, and dementia with Lewy bodies, all of which share a considerable clinical and pathological overlap^1^. α-synucleinopathies are also associated with age-related memory decline^2^; thus, there is a compelling need to understand the role of α-Syn deposition in determining the progression of the pathology and the underlying disease mechanisms.

In PD, the formation of α-Syn aggregates has been causally linked to the degeneration of dopaminergic neurons originating in the substantia nigra *pars compacta* (SNpc) and it is responsible for the onset of motor symptoms^3^. However, α-synucleinopathy also extends to other brain areas that receive dopaminergic projections from the mesencephalon, such as the hippocampus and cortex^4,5^, where it is believed to contribute to the development of early manifesting cognitive and neuropsychiatric deficits^6^. These deficits entail an impairment in working memory, language fluency, and verbal learning^7^, affecting about 40% of PD patients and representing the most debilitating non-motor symptoms and risk factors for the development of dementia in PD^8,9^. However, the heterogeneity in the onset and progression of these symptoms makes it difficult to define their relevance for an early targeted intervention aimed at delaying the onset of dementia. Notably, α-Syn aggregates have been detected post-mortem in the hippocampus of individuals with PD^10,11^ and the extent of the pathology positively correlated with the degree of cognitive impairment^12^.

Previous studies in experimental models of α-synucleinopathy showed that recombinant adeno-associated viral (rAAV) vector-mediated overexpression of human α-Syn (rAAV-hu-α-Syn) in the midbrain (ventral tegmental area/substantia nigra pars compacta, VTA/SNpc) leads to early occurrence of highly selective motor learning deficit and associated impairment of striatal metaplasticity before the onset of neuronal loss^13^. This highly selective deficit progresses towards midbrain dopaminergic (DA) denervation leading to impairment of hippocampus-dependent memory tasks and synaptic plasticity, such as long-term potentiation (LTP)^14,15^. In accordance with this, mesencephalic dopamine release regulates hippocampal-dependent memory through the activation of D1-D5 dopamine receptors, and hippocampal atrophy has been associated with deficits in recognition memory^16^ and memory-encoding processes in PD patients^17,18^.

In vivo and in vitro models of hippocampal α-synucleinopathy have shown, however, that direct overexpression or oligomerization of α-Syn impairs hippocampal synaptic plasticity, one of the neuronal correlates of memory storage and encoding, by altering the function of glutamate receptors. In particular, α-synucleinopathy impairs NMDA and AMPA-glutamate receptor-dependent LTP^19,20^.

Although these findings suggest that abnormal hippocampal aggregation of α-Syn leads to synaptic dysfunction mainly associated with altered glutamate receptor function, the disease mechanisms and associated progression of behavioral manifestations remain elusive. Furthermore, recent evidence in humans and rodents suggest that pathological deposits of α-Syn originating in different brain regions (e.g., hippocampus and caudate nucleus) lead to distinct pathologies in mice^21,22^.

Using an already established experimental model^13^, in this study we have explored the time-dependent memory deficits that emerge after hu-α-Syn overexpression in the mesencephalon and hippocampus, correlating them with the synaptic and biochemical changes occurring in the latter brain region. We report that the loss of mesencephalic DA neurons leads to *late* onset of object memory capacity deficits that are associated with a general deterioration of performance in sensorimotor and anxiety-like behavioral tests and reduced dopamine D1 receptor expression in the hippocampus. By contrast, hippocampal injections of the same vector led to *early* onset highly selective memory deficits that were associated with reduced GluA1-AMPA receptor expression and impaired hippocampal synaptic plasticity, but not with neurodegeneration, which remained stable for months.

These findings provide evidence for the pathological mechanisms and time-dependent progression of α-synucleinopathy originating in different brain regions.

## Results

### Overexpression of hu-α-Syn in the mesencephalon leads to time-dependent late onset of behavioral impairments in the motor, emotional, and memory domain associated with reduced dopamine functions

**Fig. 1:**
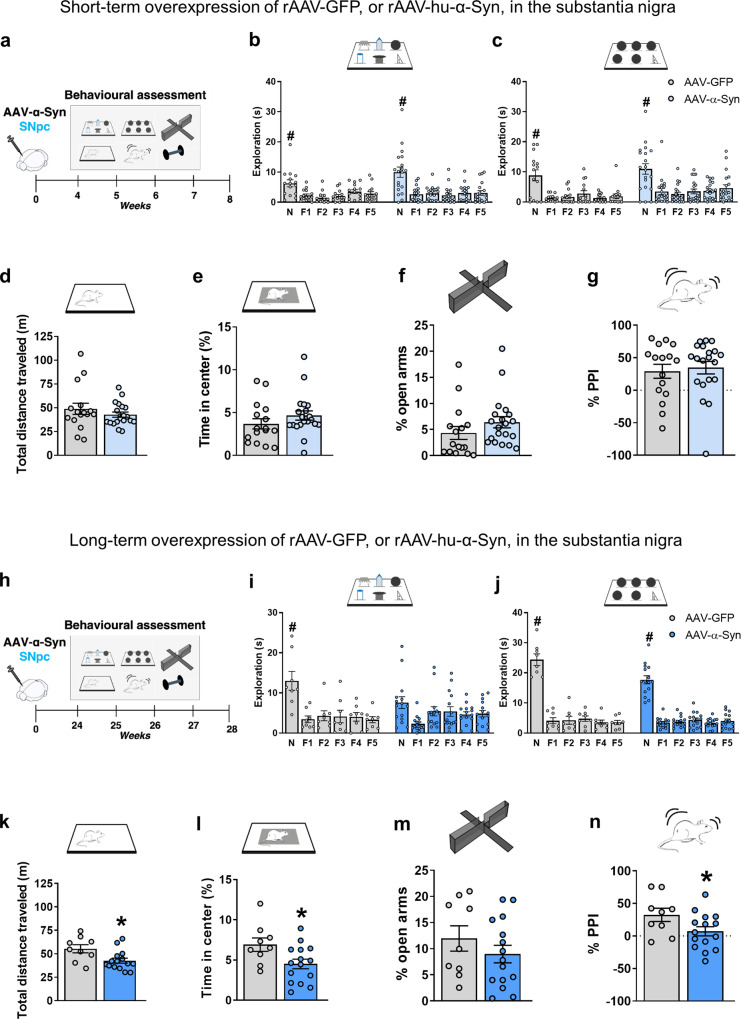
Short and long-term rAAV-mediated overexpression of GFP and hu-α-Syn in the midbrain. **a**, **h** Schematic representations of behavioral assessment at 4- and 24-weeks after injections with rAAV-hu-α-Syn or rAAV-GFP vectors. **b**, **c** SNpc-hu-α-mice compared to SNpc-GFP mice do not show differences in the exploration of the novel object (N) compared to all the familiar ones (F1-F5) in both the DOT and IOT tasks (ST rAAV-GFP 6-DOT repeated measures ANOVA: *F*(5,70) = 9.75; *P* < 0.001; ST rAAV-hu-α-Syn 6-DOT repeated measures ANOVA: *F*(5,90) = 14.99; *P* < 0.001; ST rAAV-GFP 6-IOT repeated measures ANOVA: *F*(5,70) = 12.82; *P* < 0.001; ST rAAV-hu-α-Syn 6-IOT repeated measures ANOVA: *F*(5,95) = 17.45; *p* < 0.001), (**d**, **e**) exploratory behavior in the open field or (**f**) in the elevated plus maze, (**g**) as well as in pre-pulse inhibition in the startling reflex-box. **i**–**n** In LT-experiment, rAAV-GFP explored significantly more the novel object (N) compared to all the familiar ones (F1-F5) in both the DOT and IOT tasks. However, rAAV-hu-α-Syn showed a specific impairment in recognition of the novel object in the DOT but not in the IOT task. Indeed, the novel object was not significantly more explored then all the familiars in the 6-DOT (LT rAAV-GFP 6-DOT repeated measures ANOVA: *F*(5,35) = 9.79; *P* < 0.001; LT rAAV-hu-α-Syn 6-DOT repeated measures ANOVA: *F*(5,70) = 2.94; *P* = 0.018; LT rAAV-GFP 6-IOT repeated measures ANOVA: *F*(5,35) = 102.99; *P* < 0.001; LT rAAV-hu-α-Syn 6-IOT repeated measures ANOVA: *F*(5,70) = 74.36; *p* < 0.001). **k**, **l** SNpc-hu-α-Syn mice, as compared to SNpc-GFP, also showed reduction in distance traveled and time spent in center of the open field (unpaired *t* test, *t* = 2.65, *P* < 0.05 and *t* = 2.39, *P* < 0.05, respectively, SNpc -GFP *n* = 9; SNpc -hu-α-Syn, *n* = 15), (**m**), but not in the percentage time spent in the open arm, suggesting hypokinesia and increased avoidance behavior. Finally, (**n**) SNpc-hu-α-Syn, compared to SNpc-GFP mice, showed impaired pre-pulse inhibition, an endophenotype of psychosis (unpaired *t* test, *t* = 2.08, *P* < 0.05, SNpc-GFP, *n* = 9; SNpc-hu-α-Syn, *n* = 15). Data are presented as mean ± SEM. *P < 0.05 different from SNpc-GFP control. ^#^*P* < 0.05 N different from F1–F5 (Dunnett’s test).

The rAAV2/6-hu-α-Syn vector or rAAV2/6-GFP vector was bilaterally injected into the VTA/SNpc of young adult naïve animals (here called SNpc-α-Syn mice and SNpc-GFP mice), which were then subjected to behavioral experiments 4-weeks (short-term experiment, ST) or 24-weeks (long-term experiment, LT) post-injection. A schematic representation of the experimental design is reported in Fig. 1a, h. Detailed protocols and the histological, biochemical and electrophysiological characterization of SNpc-α-Syn mice have been reported in a previous manuscript^13^. We reported there that SNpc-α-Syn mice showed impaired motor learning in the rotarod task and associated metaplasticity in the ST experiment. This highly selective motor learning deficit was not associated with DA neuronal loss, but DAT expression was reduced.

Here, we have added the results of additional behavioral tests. In the ST experiment, SNpc-α-Syn mice were not impaired in any of the behavioral tasks (Fig. 1b–g), except for the rotarod test previously reported^13^, compared to SNpc-GFP (control) mice.

By contrast, in the LT experiment, SNpc-α-Syn mice showed a significant impairment in many of the tasks performed (Fig. 1h–n). We analyzed object memory using a modified version of the object recognition task, the 6-different object task (6-DOT), that we have shown previously to be sensitive to changes in hippocampal glutamate ionotropic receptor function, as well as to systemic administration of dopamine receptor antagonists^23,24^. In the 6-DOT, mice were exposed to 6 different objects during the study phase (6-DOT, Fig. 1i), which represents a high memory load condition for mice. As a control task, we used the low memory load condition, with 6 identical objects (6-IOT, Fig. 1j). The latter controls for any unspecific effect on motivation, arousal, or motor abilities, and it does not require the functional activation of the hippocampus. In the LT-experiment, SNpc-α-Syn mice showed impaired novel object preference in the 6-DOT (Fig. 1i) but not in the 6-IOT (Fig. 1j). Notably, SNpc-α-Syn mice had reduced spontaneous locomotor activity (Fig. 1k) and spent less time in the center of an open field (Fig. 1l). No changes were detected in the time spent in the open arms of an elevated plus maze (Fig. 1m). In addition, SNpc-α-Syn mice showed reduced percentage of pre-pulse inhibition (PPI) compared to SNpc-GFP mice (Fig. 1n). This last measure is used to model positive symptoms of psychosis in mice.

These results suggest that long-term overexpression of hu-α-Syn in the SNpc affects multiple cognitive and behavioral domains, triggering memory deficits, increased thigmotaxis, and inducing sensorimotor gating abnormalities.

### Late onset memory deficits induced by overexpression of hu-α-Syn in the mesencephalon are associated with reduced D1 dopamine receptor expression in the hippocampus

**Fig. 2:**
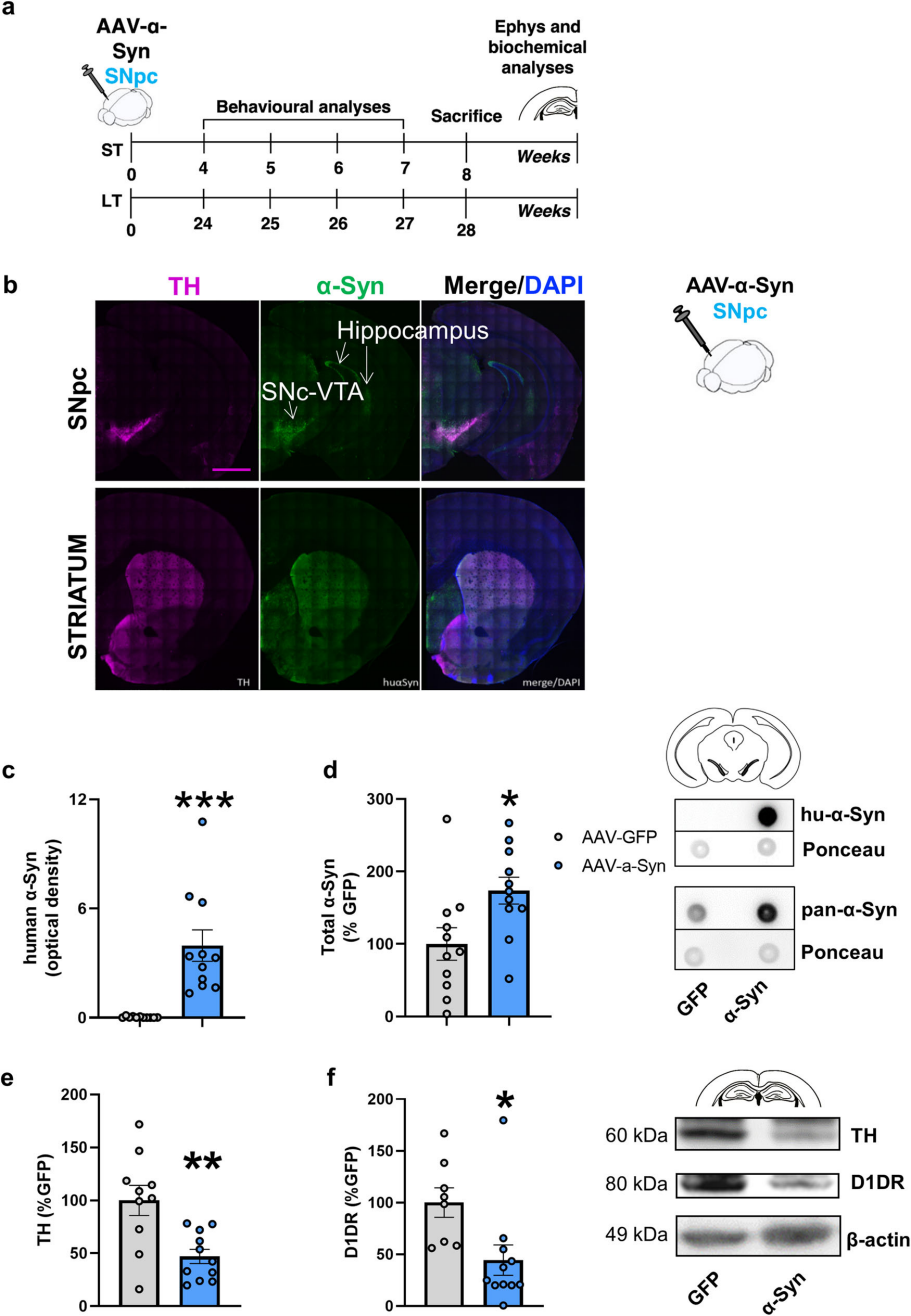
rAAV-mediated overexpression of hu-α-Syn in the midbrain leads to reduced TH and D1 receptor expression in the hippocampus. **a** Schematic representation of the experimental design for the short-term and long-term experiments, including behavioral testing starting 4- or 24-weeks after intra-SNpc injections as described in^13^. After behavioral testing animals were sacrificed for immunohistochemical, biochemical and ex-vivo electrophysiological analysis (for the short-term time point). **b** Representative coronal section of hemibrain of SNpc-hu-α-Syn mice showing wide expression of hu-α-Syn in the target regions of the VTA/SNpc, including the hippocampus (dorsal and ventral), the septum and the striatum (Scale bar = 1 mm). Detailed histological analysis of this group of animals has been previously reported in ref. ^13^. **c**, **d** Dot-blot analysis of hu-α-Syn (unpaired *t* test, *t* = −4.55, *P* = 0.0002, SNpc-GFP, *n* = 11; SNpc -hu-α-Syn, *n* = 11) and of pan-α-Syn expression (unpaired *t* test, *t* = −2.53, *P* = 0.019, SNpc-GFP, *n* = 11; SNpc -hu-α-Syn, *n* = 11) in mesencephalic tissue of SNpc-hu-α-Syn mice and SNpc-GFP mice. **e**, **f** The overexpression of hu-α-Syn in the SNpc/VTA significantly decreased the expression of TH (unpaired *t* test, *t* = 3.451, *P* = 0.0027, SNpc-GFP, *n* = 10; SNpc-hu-α-Syn, *n* = 11) and D1 dopamine receptor (unpaired *t* test, *t* = 2.647, *P* = 0.0169, SNpc-GFP, *n* = 8; SNpc-hu-α-Syn, *n* = 11) in the hippocampus. Representative bands for each condition are reported. Data are presented as mean ± SEM. *P < 0.05, **P < 0.01, ***P < 0.001 different from SNpc-GFP.

We have previously reported the histochemical and biochemical analysis of the brain tissues of mice with midbrain overexpression of hu-α-Syn and showed that they exhibit Tyrosine Hydroxylase positive (TH^+^) neuronal loss in the mesencephalon and reduced striatal TH protein expression in the LT but not in the ST experiments (as schematized in Fig. 2a and reported in ref. ^13^). Thus, the onset of memory deficits in the LT experiment might be due to reduced DA signaling in the striatum in this group of animals (Supplementary Fig. 1c).

However, after hu-α-Syn overexpression in the mesencephalon, axonal transgene expression is present in many other target regions of the mesencephalon, including the hippocampus (Fig. 2b). Therefore, in this study, we extended our previous observations by further confirming, using dot-blot analysis, the expression of hu-α-Syn in the SNpc (Fig. 2c). A pan-α-Syn antibody, which detects both human and mouse protein, showed an approximate 70% increase of total α-Syn in hu-α-Syn overexpressing mice, compared to GFP (Fig. 2d). To correlate the 6-DOT memory impairment observed in SNpc-hu-α-Syn mice in the LT experiment with biochemical changes in the hippocampus, we performed Western blot analysis on their hippocampi and found a significant reduction in TH protein expression (Fig. 2e), which was associated with reduced expression of D1 dopamine receptor (D1DR) (Fig. 2f). Both results were confirmed by immunofluorescence analysis of TH immunoreactivity (Supplementary Fig. 1a) and D1DR spots (Supplementary Fig. 1b). A reduction in TH immunoreactivity was also observed in the striatum of SNpc-hu-α-Syn mice (Supplementary Fig. 1c).

### Overexpression of hu-α-Syn in the dorsal hippocampus leads to early selective memory capacity loss and associative learning deficits that remain stable over time

**Fig. 3:**
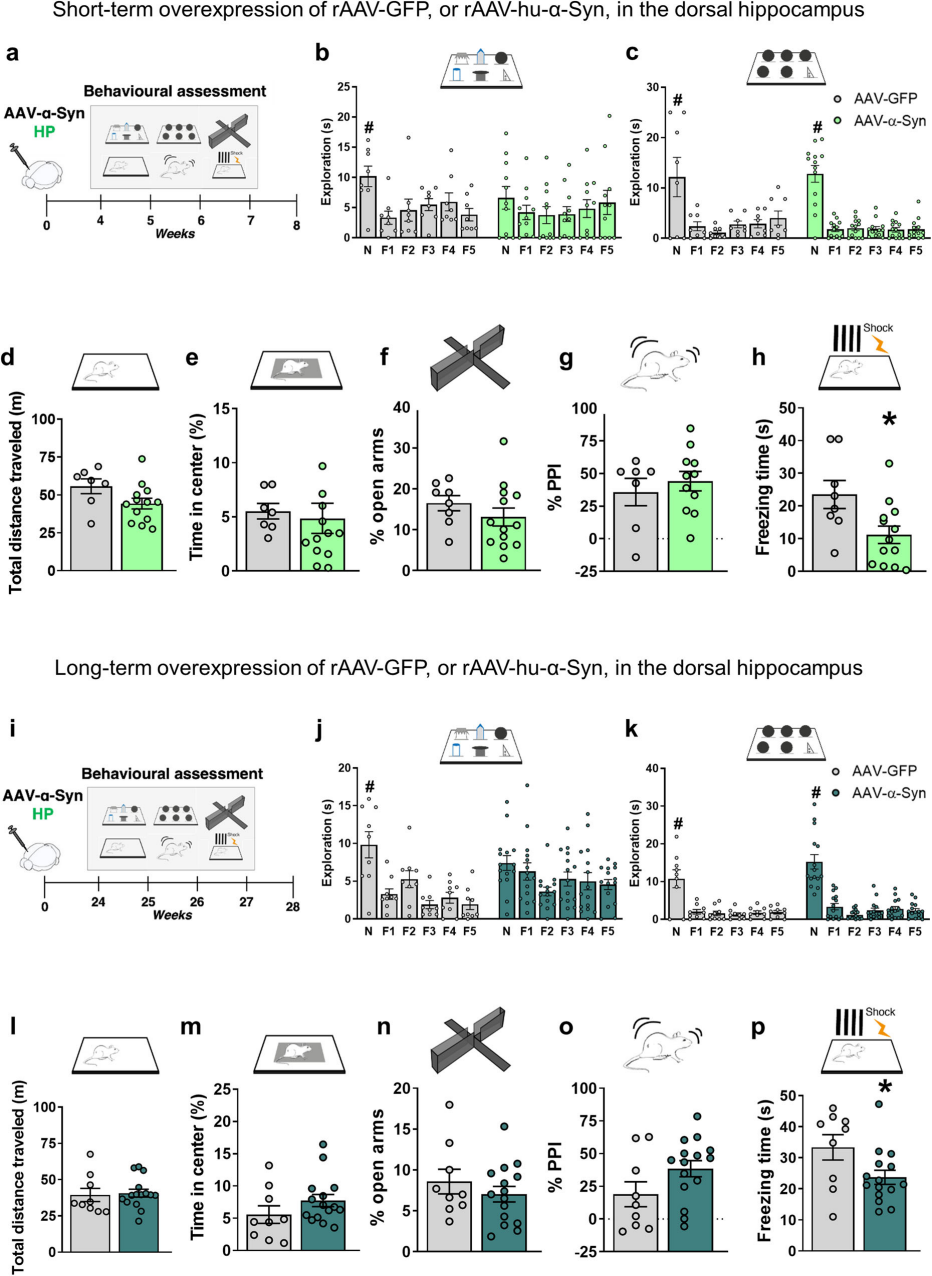
Short and long-term rAAV mediated overexpression of GFP and hu-α-Syn in the dorsal hippocampus. **a**, **i** Schematic representations of behavioral assessment at 4- and 24-weeks after injections with rAAV-hu-α-Syn or rAAV-GFP vectors. **b**, **c** Short-term rAAV-GFP injected animals explored significantly more the novel object (N) compared to all the familiar ones (F1–F5) in both the DOT and IOT tasks, while hu-α-Syn injected animals showed impairment in the 6-DOT but not in 6-IOT (ST rAAV-GFP 6-DOT repeated measures ANOVA: *F*(5,35) = 5.23; *P* = 0.0011; ST rAAV-hu-α-Syn 6-DOT repeated measures ANOVA: *F*(5,50) = 0.974; *P* = 0.44; ST rAAV-GFP 6-IOT repeated measures ANOVA: *F*(5,35) = 6.74; *P* < 0.001; ST rAAV-hu-α-Syn 6-IOT repeated measures ANOVA: *F*(5,60) = 47.59; *P* < 0.001). **d** No significant changes in measures of exploratory behavior, such as the distance traveled in the open field or (**e**) percentage of time spent in the center of the arena, (**f**) percentage of time in the open arm in the plus maze task, and (**g**) in the pre-pulse inhibition were observed in HP-hu-α-Syn mice when compared to GFP. In contrast, (**h**) HP-hu-α-Syn mice show a significant reduction in freezing time, during the testing day, in the contextual fear-conditioning task, suggesting the presence of highly specific memory impairment (Group, *F*(1,19) = 6.67, *P* < 0.05, Time, *F*(5, 95) = 8.44, *P* < 0.001, Group × Time, *F*(5,95) = 5.19, *P* < 0.001). **j** Long-term overexpression of hu-α-Syn in the dorsal hippocampus leads to impairing effects identical to the short-term expression in the novel object exploration in conditions of high load (LT rAAV-GFP 6-DOT repeated measures ANOVA: *F*(5,40) = 9.87; *P* < 0.001; LT rAAV-hu-α-Syn 6-DOT repeated measures ANOVA: *F*(5,70) = 2.86; *P* = 0.02; LT rAAV-GFP 6-IOT repeated measures ANOVA: *F*(5,40) = 16.92; *P* < 0.001; LT rAAV-hu-α-Syn 6-IOT repeated measures ANOVA: *F*(5,70) = 48.0; *P* < 0.001), and (**p**) in the freezing time during the fear-conditioning test (Group, *F*(1,22) = 5.05, *P* < 0.05: Time, *F*(5, 110) = 6.42, *P* < 0.001), (**k**–**o**) without affecting performance in any of the other behavioral tests. Data are presented as mean ± SEM. *P < 0.05 different from HP-GFP control. ^#^*P* < 0.05 N different from F1-F5 (Dunnett’s test).

The progressive memory impairment observed after midbrain overexpression of hu-α-Syn manifested only several months after the injection and was associated with other behavioral deficits. To test if this was the consequence of a selective hippocampal hu-α-Syn overexpression, we tested the effect of bilateral hippocampal hu-α-Syn overexpression by injection of rAAV2/6-hu-α-Syn or rAAV2/6-GFP vectors in naïve mice (here called HP-hu-α-Syn mice and HP-GFP mice) in ST and LT experiments (Fig. 3a, i). Results showed that HP-hu-α-Syn mice had decreased novel object preference in the 6-DOT (Fig. 3b), but not in the 6-IOT (Fig. 3c), when compared to HP-GFP mice. The control and injected groups showed similar object exploration during the study phase (data not shown), suggesting that the defect was associated with impaired memory in high-load conditions rather than a general reduction in exploration. In addition, the deficit in object memory was not driven by changes in motor activity or thigmotaxis. Indeed, no changes were detected in spontaneous locomotor activity (Fig. 3d), time spent in the center of an open field (Fig. 3e), percentage of time spent in the open arms during elevated plus maze test (Fig. 3f), and in sensorimotor gating in the acoustic startle reflex box (Fig. 3g). Furthermore, the specific impairment in hippocampal functions was also demonstrated in the contextual fear conditioning test. Specifically, HP-hu-α-Syn mice showed a marked reduction in freezing time (Fig. 3h). During training, no differences were detected between HP-GFP and HP-hu-α-Syn mice (13.01 s ± 3.87 S.E.M. vs. 15.77 s ± 5.10 S.E.M, respectively), indicating that hippocampal overexpression of hu-α-Syn leads to deficits in associative long-term memory. Together, these findings show that rAAV vector-mediated hu-α-Syn overexpression in the dorsal hippocampus leads to highly specific memory deficits in the ST experiment.

To study the long-term effects of hu-α-Syn overexpression (LT experiment) in the dorsal hippocampus, we performed a separate set of experiments in which behavioral tests were performed 24 weeks after the viral vector injection. HP-hu-α-Syn mice showed decreased novel object preference in the 6-DOT (Fig. 3j), but not in the 6-IOT (Fig. 3k), compared to HP-GFP mice. HP-hu-α-Syn mice did not show altered spontaneous locomotor activity (Fig. 3l), time spent in the center of an open field (Fig. 3m), percentage of time spent in the open arms of an elevated plus maze test (Fig. 3n), and percentage of PPI (Fig. 3o). As observed 4 weeks post-injection, HP-hu-α-Syn mice showed reduced freezing time in the fear conditioning test (Fig. 3p) and no differences during the training phase compared to HP-GFP mice (58.77 s ± 12.79 S.E.M. vs. 58.64 s ± 9.77 S.E.M, respectively).

Together, these data suggest that memory alterations induced by hippocampal overexpression of hu-α-Syn persist unaltered for a long period of time and that the pathology does not affect non-memory related domains.

### rAAV vector-mediated hu-α-Syn overexpression in the hippocampus leads to early reduced GluA1 expression and impaired synaptic plasticity

**Fig. 4:**
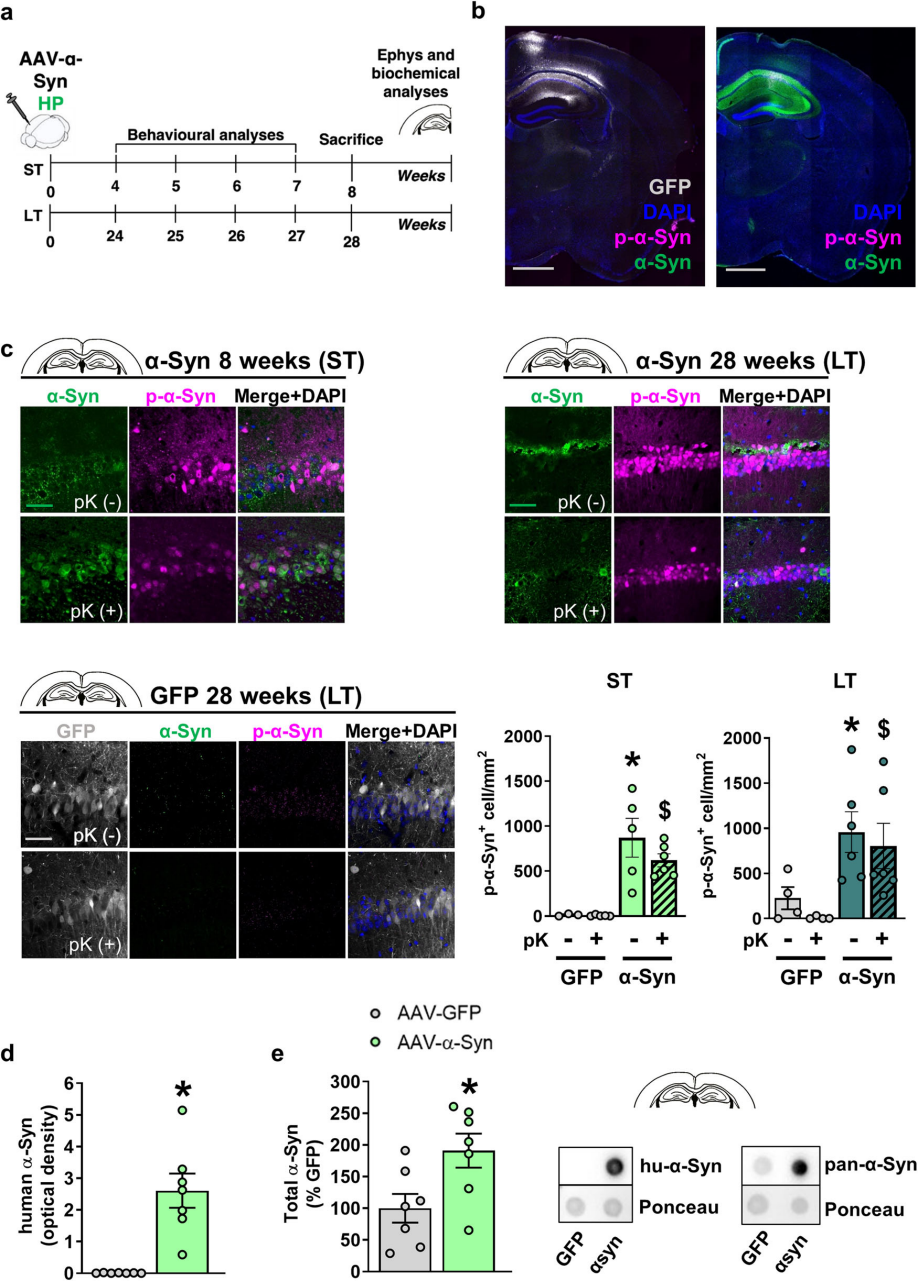
rAAV vector-mediated expression of hu-α-Syn in the dorsal hippocampus leads to sustained overexpression of pK-resistant α-Synuclein. **a** Schematic representation of the experimental design for the short-term and long-term experiments, including behavioral testing starting 4- or 24-weeks after intra-brain injection and lasting for about 4 weeks. After behavioral testing, animals were sacrificed for immunohistochemical, biochemical and ex-vivo electrophysiological analysis (for the short-term time point)**. b** Representative sagittal brain section (mosaic reconstruction) of the injection placement of rAAV-GFP (left panel) and rAAV-hu-α-Syn (right panel) vectors, showing a wide distribution of GFP and hu-α-Syn in the whole hippocampus (Scale bar: 1 mm). **c** Representative confocal images showing α-Syn (green) and p-α-Syn (magenta) expression in the CA1 of the HP injected with rAAV-hu-α-Syn or r-AAV-GFP, in basal immunostaining conditions (pK^-^) and after the treatment with proteinase K (pK^+^), for mice sacrificed at 8 weeks (ST) or 28 weeks (LT) after intra-brain injection (Scale bar: 50 μm). Confocal images at the bottom show undetectable α-Syn (green) and p-α-syn (magenta) expression in the CA1 of the HP of an rAAV-GFP mouse sacrificed at 28 weeks after injection, under basal conditions and after treatment with pK (Scale bar: 50 μm). Quantitation of the number of p-α-Syn positive cells (p-α-Syn^+^ cell/mm^2^) showed a significant effect for vector injection in the ST experiment (Injection, *F*(1,14) = 29.244, *P* < 0.0001, pK treatment, *F*(1,14) = 0.890, *P* = 0.3615, Injection × pK treatment, *F*(1,14) = 0.811, *P* = 0.3830), and a significant difference between rAAV-GFP and rAAV hu-α-Syn pK^-^ (unpaired *t* test, *t* = 2.978, *P* = 0.0247, rAAV-GFP, *n* = 3; rAAV-hu-α-Syn, *n* = 5) and pK^+^ (unpaired *t* test, *t* = 6.789, *P* = 0.0001, rAAV-GFP, *n* = 5; rAAV-hu-α-Syn, *n* = 6). A similar effect was found in the LT experiment (Injection, *F*(1,16) = 12.433, *P* = 0.0028, pK treatment, *F*(1,16) = 0.728, *P* = 0.4062, Injection × pK treatment, *F*(1,16) = 0.022, *P* = 0.8846), again with a significant difference between rAAV-GFP and rAAV hu-α-Syn pK^-^ (unpaired *t* test, *t* = 2.448, *P* = 0.0401, rAAV-GFP, *n* = 4; rAAV-hu-α-Syn, *n* = 6) and pK^+^ (Mann–Whitney test, *P* = 0.0095, rAAV-GFP, *n* = 4; rAAV-hu-α-Syn, *n* = 6). *P < 0.05 vs. HP-GFP pK^-^. ^$^*P* < 0.05 vs. HP-GFP pK^+^. **d**, **e** Dot-blot analysis of hu-α-Syn (unpaired *t* test, *t* = −4.81, *P* = 0.0004, rAAV-GFP, *n* = 7; rAAV-hu-α-Syn, *n* = 7) and pan-α-Syn expression (unpaired *t* test, *t* = −2.58, *P* = 0.023, rAAV-GFP, *n* = 7; rAAV-hu-α-Syn, *n* = 7) in hippocampal tissue of HP-hu-α-Syn and HP-GFP mice. Data are presented as mean ± SEM. *P < 0.01 different from HP-GFP.

We characterized the histological effects of rAAV-hu-α-Syn vector in the hippocampus (Fig. 4a). The HP-hu-α-Syn mouse brain showed high expression of hu-α-Syn in all *cornu ammonis* subregions (Fig. 4b). Phosphorylation of α-Syn at residue S129 (here defined as p-α-Syn) occurs subsequent to initial α-Syn aggregation and thus is considered a pathological marker. In the hippocampus of HP-hu-α-Syn mice, p-α-Syn was mainly detected around the injection site (Fig. 4b, right panel and Fig. 4c, top panels). Control mice injected with the rAAV-GFP showed a similar spread of GFP across the *cornu ammonis* subregions but almost no detectable expression of p-α-Syn (Fig. 4b, left panel and Fig. 4c, bottom left panel), suggesting that increased p-α-Syn expression at the injection site was due to hu-α-Syn overexpression. Using a proteinase K (pK) digestion protocol that detects insoluble forms of α-Syn that are resistant to the treatment^25^, we found that AAV-mediated hu-α-Syn overexpression led to the formation of pK-resistant forms of α-Syn in both ST and LT experiments (Fig. 4c). Hu-α-Syn overexpression was also confirmed by dot-blot analysis in the hippocampus (Fig. 4d). Moreover, the pan-α-Syn antibody revealed an approximately 90% increase of total α-Syn in HP-hu-α-Syn overexpressing mice, compared to GFP (Fig. 4e).

A pathological role of α-Syn aggregates on the activity of the autophagy/lysosomal system, an essential catabolic process involved in the degradation of misfolded proteins^26^, has been demonstrated in several studies^27,28^. Also, stimulation of autophagy has been shown to rescue motor and memory deficits in animal models of α-synucleinopathy^2,3,29,30^. For these reasons, we decided to assess the effect of hu-α-Syn-overexpression on autophagy proteins LC3-II and its substrate p62. Despite massive accumulation of hu-α-Syn, LC3-II (Supplementary Fig. 2a) and p62 (Supplementary Fig. 2b) were not affected in hu-α-Syn overexpressing hippocampi. In addition, cell counting of pyramidal neurons (NeuN^+^) did not reveal neuronal loss at any of the time-points tested (Supplementary Fig. 3a–c). These findings suggested that injection of the rAAV-hu-α-Syn vector led to α-Syn accumulation, but this was not sufficient to induce neuronal loss and thus the impairment was likely at the synaptic level.

**Fig. 5:**
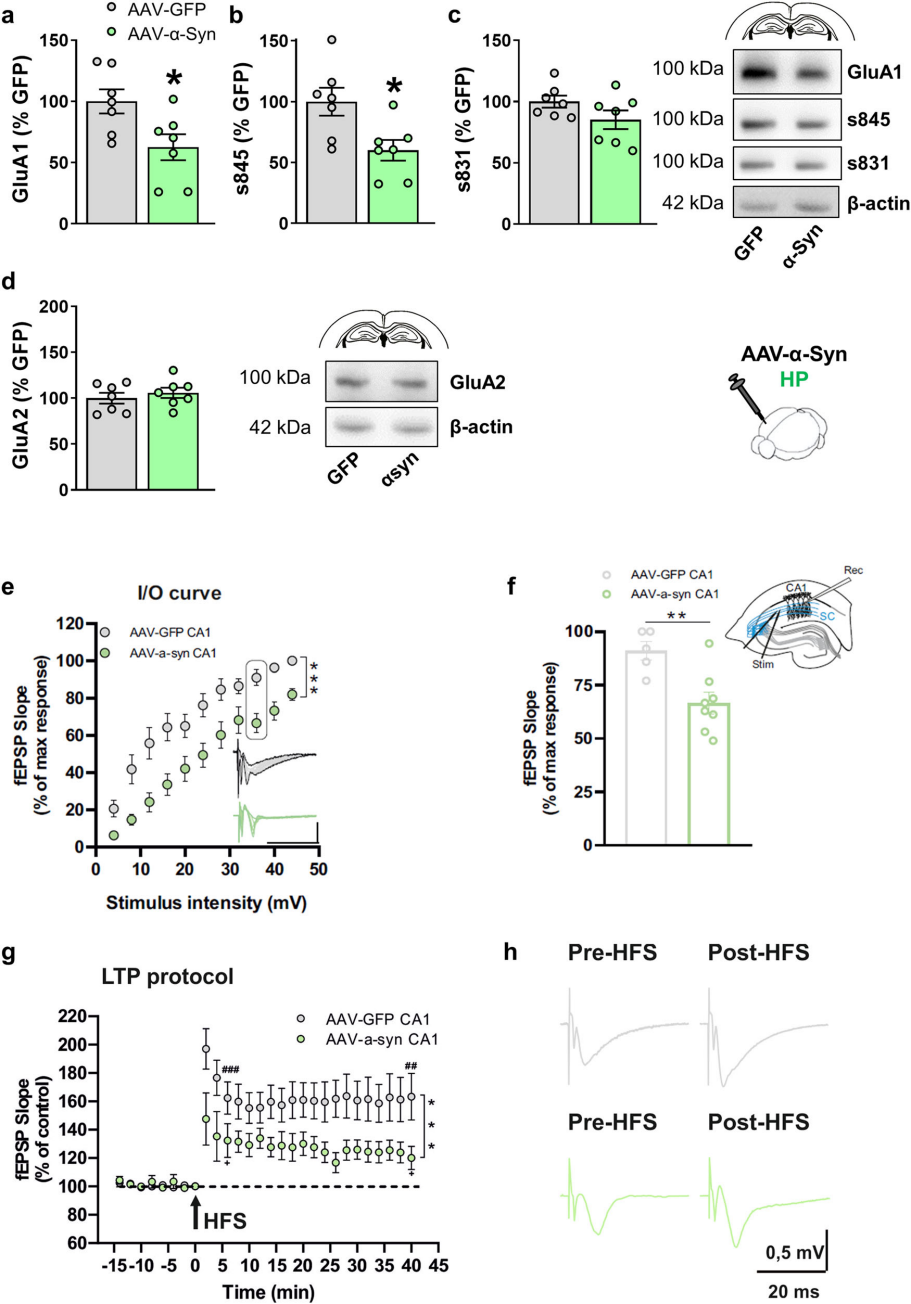
Short-term overexpression of hu-α-Syn in the hippocampus leads to reduced GluA1 AMPA receptors expression and phosphorylation associated with impaired long-term potentiation. **a** Western blot analysis of the hippocampal tissue showing that administration of rAAV-hu-α-Syn vector leads to reduced expression of GluA1 (unpaired *t* test, *t* = 2.582, *P* = 0.02, HP-GFP, *n* = 7; HP-hu-α-Syn, *n* = 7) and (**b**) serine 845 phosphorylation (unpaired *t* test, *t* = 2.807, *P* = 0.01, HP-GFP, *n* = 7; HP-hu-α-Syn, *n* = 7) (**c**) but not serine 831 phosphorylation. **d** Intra-hippocampal overexpression of rAAV-hu-α-Syn did not affect the expression of the GluA2 subunit. Representative bands for each condition are reported. β-actin reported in 5d belong to the same blot reported in Supplementary Fig. 2a. **e**, **f** Field EPSPs input–output curves revealed a significant decrease in excitability in the HP-hu-α-Syn mice with respect to HP-GFP mice (two-way ANOVA: HP-hu-α-Syn *n* = 11, HP-GFP *n* = 11, F (11,220) = 5.090, Bonferroni’s post hoc test ***P < 0.001). **g** High-frequency stimulation (HFS)-induced LTP is reduced in hippocampal slices of HP-hu- α-Syn mice (green circles), compared to the HP-GFP group (gray circles) (two-way ANOVA: HP-hu-α-Syn *n* = 8, HP-GFP *n* = 11, *F*(23,391) = 2.690, Bonferroni’s post hoc test ***P < 0.001). Paired Student *t* test 2 min pre vs. 6 min post HFS: rAAV-GFP *n* = 11, *t* = 5.674, df = 10, ^###^*P* < 0.001; rAAV-hu-α-Syn *n* = 8, *t* = 2.749, df = 7, ^+^*P* < 0.05; 2 min pre vs. 38 min post HFS: rAAV-GFP *n* = 11, *t* = 3.995, df = 10, ^##^*P* < 0.01; rAAV-hu-α-Syn *n* = 8, *t* = 2.450, df = 7, ^+^*P* < 0.05. **h** Representative traces of evoked fEPSPs recorded before and 40 min after a high-frequency stimulation (HFS) protocol in HP-GFP CA1 (gray traces) and HP-α-Syn CA1 (green traces) animals. Data are presented as mean ± SEM. *P < 0.05 different from HP-GFP.

To address whether proteinopathy affected synaptic protein expression, we performed Western blot analysis of hippocampi of HP-hu-α-Syn mice compared to HP-GFP mice. Since the memory impairment was already evident in mice subjected to the short-term experiment, and it was not associated with TH^+^ neuronal loss after midbrain injection^13^, we focused our analysis on glutamate AMPA and NMDA receptors. Hippocampi of HP-hu-α-Syn mice showed reduced expression of GluA1 (Fig. 5a) and reduced phosphorylation at s845 (Fig. 5b), but not at s831 (Fig. 5c). Reduced GluA1 expression was long-lasting (Supplementary Fig. 3d, e). This defect was highly specific as no significant changes were observed in NMDA receptor subunit expression (Supplementary Fig. 4a–c) or in the expression of other post-synaptic proteins or proteins of the SNAP receptor complex (SNARE) (Synapsin 1/2, SNAP-25, Snapin, VAMP2, Synaptophysin, vGlut1, CSPα, and PSD-95 Supplementary Fig. 5a–h) or GluA2 (Fig. 5d).

To verify if the reduced expression of GluA1 and its phosphorylated forms was associated with altered synaptic glutamatergic response and hippocampal plasticity, further groups of HP-hu-α-Syn and HP-GFP mice were used for ex-vivo electrophysiological analysis. Field excitatory postsynaptic potentials (fEPSPs) were recorded in order to obtain input–output (I/O) curves. Variation in the fEPSPs slope in response to increasing stimulus intensities was observed in both groups. However, the response amplitudes measured in HP-hu-α-Syn mice with respect to HP-GFP were significantly reduced, revealing a significant change in excitability in this experimental group (Fig. 5e, f). Moreover, LTP was significantly reduced in hippocampal slices from HP-hu-α-Syn mice compared to HP-GFP mice (Fig. 5g, h), as revealed by decreased expression and phosphorylation of the GluA1 AMPA subunit (Fig. 5a, b). Indeed, the analysis of the fEPSP slope between the baseline and two time points after HFS application showed a reduction of LTP amplitude in the HP-hu-α-Syn versus HP-GFP mice (Fig. 5g, h).

In addition, in LT experiments, these highly specific memory defects were associated with reduced GluA1 expression in the hippocampus (Supplementary Fig. 3d, e). In contrast, no changes in GluA1 expression or phosphorylation were observed in the hippocampus of SNpc-hu-α-Syn mice in the LT-experiment (Supplementary Fig. 6a–c).

Together, these data suggest that hippocampal overexpression of hu-α-Syn has a specific impact on GluA1 expression and phosphorylation, and results in local pyramidal neuron connectivity impairment.

## Discussion

In the present study we report that rAAV-mediated human-α-Syn overexpression is responsible for brain region-specific onset and progression of behavioral dysfunctions. In particular, when α-Syn overexpression-induced proteinopathy originates in the midbrain it leads to a time-dependent late onset of memory deficits that is accompanied by generalized behavioral dysfunctions, such as bradikynesia, increased anxiety-like behavior, and impaired sensorimotor gating. This overexpression also correlates with reduced DA signaling, not only in the striatum as previously reported^13^, but also in the hippocampus, and in particular on D1 dopamine receptors. In contrast, when α-Syn overexpression-induced proteinopathy originates in the dorsal hippocampus, it leads to early selective memory impairments that are not associated with changes in autophagy protein levels and neurodegeneration, but rather are related to the downregulation of GluA1 receptor expression. Importantly, these early memory defects remain stable over months, and do not affect other behavioral domains. Taken together, these findings indicate that the different progression of memory deficits and associated hippocampal disease mechanisms in response to increased α-Syn depend on the specific brain site of onset.

rAAV-α-Syn overexpression in the midbrain leads to akinesia, increased anxiety-like behavior, reduced sensorimotor gating, and onset of memory deficits in the 6-DOT. This type of object memory deficit adds to the previously demonstrated visuospatial memory impairment reported in PD patients and in animal models of PD^31,32^. These observations provide a full characterization of the time-dependent behavioral deficits in this model, and report non-motor deficits, e.g., impaired pre-pulse inhibition, that are considered as endophenotypes of psychosis and are still little explored in animal models of α-synucleinopathy. These findings have high relevance for translational research aimed at identifying targeted antipsychotic and anxiolytic drugs for the treatment of PD and dementia with Lewy bodies^33^. Based on the histological characterization of these animals^13^ and on the reduced TH expression in the dorsal hippocampus of SNpc-hu-α-Syn mice reported here, the onset of these deficits might be traced back to DA denervation manifesting at late stages and/or to the synaptic impairments induced by α-Syn reaching the hippocampus from the mesencephalon. In addition, although no change in GluA1 expression was observed, we found reduced expression of D1 receptors, which are fundamental for the formation and maintenance of different types of hippocampal-dependent memory^16^.

The progression of the behavioral deficits observed in HP-hu-α-Syn mice differs from that observed in SNpc-hu-α-Syn mice. The early synaptic and behavioral impairments induced by hippocampal overexpression of human α-Syn remained stable over 24-28 weeks post-injection, as evidenced by the lack of reduction in NeuN-expressing cells. Our findings do not exclude the possibility that adding a later time point post-injection might reveal hippocampal neuronal loss; however, in this experimental model, 28 weeks are sufficient for DA neuronal loss in the SNpc^13^, as well as reduced TH expression in the striatum, thereby confirming a high vulnerability of DA neurons to α-Syn overexpression as compared with pyramidal cells.

A recent study combining unilateral rAAV-mediated overexpression of α-Syn in the SNpc and in the hippocampus reported neuronal loss at 16 weeks post-injection only in the former region^34^. Together with our observations, this suggests that simultaneous overexpression of α-Syn in these two connected areas might accelerate DA neuronal loss through the concurring actions of pathological loads of α-Syn both in the terminal regions and in the cellular bodies of DA neurons. Whether this would also result in degeneration of hippocampal pyramidal cells at later time points remains to be established. Additionally, the same study reported early onset of memory and motor deficits, which in our bilateral model might result in an anticipation of the deficits in motor learning and object memory preceding bradykinesia, in line with the symptomatologic progression observed in PD patients.

Though these findings are consistent with the aforementioned animal models of genetically constitutive overexpression of α-Syn, direct injection of human recombinant α-Syn preformed fibrils (PFFs) has been reported to lead to pyramidal neuronal loss in the hippocampus, with differences in hippocampal subfields^35^. This suggests the hypothesis that different aggregational states of α-Syn might underlie the heterogeneity observed in human studies, which report both the presence and the absence of neuronal loss in the hippocampus of PD patients, with no statistical difference with healthy controls^8^. When α-synucleinopathy originates in the hippocampus, the substantial increase in the number of fibrils not only leads to neurodegeneration of pyramidal neurons, but also spreads across different brain regions^36^. A recent study reported that the brain-site specific vulnerability to α-synucleinopathy depends on a combination of the concentration of endogenous α-Syn and brain connectomics, supporting the model of a prion-like spreading of pathologic α-Syn^21^. Thereupon, it is tempting to speculate that PFF injection in the hippocampus might lead to time-dependent onset of behavioral deficits in different memory and behavioral domains, due to its widespread action in multiple brain regions^21^.

The early occurring memory deficit reported in HP-hu-α-Syn mice remains stable both within and between behavioral domains. In the LT experiment, object memory is still impaired in the 6-DOT, but not in the 6-IOT, and performance in all the other tests is intact. Intriguingly, these findings contrast with our recent evidence that identified early manifesting α-synucleinopathy in a spontaneous early-aging model, showing impaired performance in the 6-DOT and impaired hippocampal GluA1 signaling^2^. In this early aging model, memory capacity worsened with time (6 months later), and the performance in the 6-IOT was also affected. A possible explanation for this discrepancy could be that hippocampal α-synucleinopathy in the spontaneous aging model is associated with a block of autophagy/lysosomal function. Clearance and turnover of misfolded proteins relies on the degradative capacity of the cells^26^. Recent studies in aging have suggested that impaired activity of lysosomal genes, such as *GBA1*, does not induce α-synuclein pathology by itself, although they amplify the pathogenic effects of α-Syn^37^. In the rAAV-α-Syn vector overexpression model we did not detect impaired autophagy, despite the 70-90% increase in α-Syn expression. Thus, these findings support the view of cell-autonomous mechanisms leading to neuronal death^38^ and suggest that impaired autophagy/lysosomal function might be one of the risk factors for deterioration of cognitive performance and the severity in α-synucleinopathy^37^.

Accumulation of Lewy pathology in CA2 has been reported to correlate with the severity of cognitive impairment in PD patients^8,39^. Neuritic and synaptic dysfunction positively correlate with higher constitutive expression of the α-Syn gene SNCA, as well as other genes related to synaptic functions^22^. In particular, converging in vitro and in vivo evidence suggest that the synaptic and neurite dysfunctions provoked by α-synucleinopathy in the hippocampus are due to changes in synaptic glutamate function and synaptic organization of ionotropic glutamate receptors. Impaired NMDA receptor function has been previously reported independently on the synucleinopathy model^40–42^, and both increased and decreased GluA1 AMPA receptor expression have been reported in different experimental models^19,43,44^. Burdening hippocampal neurons with PFFs affects proteins forming the SNARE complex, leading to progressive impairment of neuronal excitability and connectivity without altering expression of GluA1 receptors^36^. However, it is well recognized that the injection of α-Syn PFFs and the overexpression of α-Syn mediated by rAAV produce different patterns of PD pathology in rodents^45^. By overexpressing α-Syn in vivo, we did not detect any change in the expression of SNARE proteins, although we found a selective reduction in the expression of GluA1 receptors and the phosphorylation of s845, which is in agreement with our previous findings in aging mice showing impaired performance in the 6-DOT, hippocampal accumulation of p-α-Syn, and reduced GluA1 function^2^. Previous in vitro evidence reported that glutamatergic synapses upon oligomeric α-Syn treatment, but not under monomeric or PFFs α-Syn, lose the ability to respond to NMDA-dependent theta-burst stimulation (TBS)-induced LTP^19^, which requires transient insertion of calcium-permeable AMPA receptors into the postsynaptic membrane. In this study, while we did not find a significant change in NMDA receptor subunit expression upon overexpression of α-Syn, reduced expression of GluA1 and its phosphorylation at serine site was observed. Since GluA1 phosphorylation at s845 regulates AMPA receptor insertion at the synapse, which is necessary for the induction and stabilization of LTP^23,46^, these findings suggest that the impairment in LTP we observed might be due to an alteration of these converging synaptic mechanisms.

The mechanisms by which α-Syn overexpression leads to reduced GluA1 expression remains to be clarified. A recent study involving quantitative proteomic analysis of hippocampal CA2 samples from PD and non-PD subjects reported specific downregulation of the multidomain neuronal scaffold proteins Caskin1 and Caskin2, which in PD is not associated with volumetric, neural, or glial changes^47^. Genetic deletion of Caskin proteins in mice has also been reported to impair the phosphorylation of AMPA receptors and the induction of LTP in hippocampal slices^48^.

The α-synucleinopathy models described here provide the basis to explore these mechanisms in future studies.

In conclusion, early memory deficits are associated with PD and dementia with Lewy bodies and they are highly regarded not only for their prognostic value for the onset of dementia, but also as early symptoms to target with restorative therapeutic strategies. However, not all early memory deficits progress to dementia. Sometimes they disappear and sometimes they worsen, albeit always remaining restricted to the memory domain. Probing the mechanisms regulating these processes is crucial to understand the nature and progression of early cognitive symptoms. In this study we provide evidence that, when α-synucleinopathy originates in the hippocampus and is due to increased expression of human wild-type α-Syn, it leads to early onset synaptic and memory defects that remain stable over time. Conversely, when α-synucleinopathy originates in the midbrain, it is associated with specific sensorimotor deficits and later converts into generalized motor and non-motor symptoms. Interestingly, hippocampal and midbrain origins of α-synucleinopathy lead to different biochemical imbalances, affecting glutamate AMPA and dopamine D1 receptor expression, respectively. From an indirect comparison with what is observed in aging, as well as after overexpression of the human mutated A53T α-synuclein^49^, the co-existence of other risk factors, including reduced autophagy/lysosomal degradative capacity, might be critical for determining whether increased expression of α-synuclein-induced mild cognitive impairment will remain stable or will worsen with time.

## Methods

### Animals

Experiments were performed in CD1 outbred male mice, 9–10 weeks old at the beginning of experiments. Animals were group-housed (Charles River), with *ad libitum* access to food and water during a 12 h light/dark cycle. Testing was performed during the light phase. All procedures relating to animal care and treatments conformed to the guidelines and policies of the European Communities Council and were approved by the Italian Ministry of Health. The number of animals used in this study is reported in Supplementary Table 1 (Table S1).

### Recombinant adeno-associated viral vector (rAAV)

The human-α-Synuclein adeno-associated viral (rAAV2/6-hu-α-Syn) vector and GFP viral (rAAV2/6-GFP) vector as control treatment were kindly provided by Professor Anders Björklund, and were those used in previous studies^3,13,50,51^. The expression of the transgene was driven by the synapsin-1 promoter and enhanced using a woodchuck hepatitis virus post-transcriptional regulatory element (WPRE).

Briefly, transfer plasmids carrying AAV2 ITRs coding for either a human-alpha-Synuclein or enhanced GFP, downstream to a synapsin-1 promoter, were generated. The transfection into HEK 293 cells was carried out using the calcium phosphate method, and included the packaging plasmids pDP6 encoding AAV6 capsid proteins. The cells were treated with a lysis buffer (50 mM Tris, 150 mM NaCl, pH 8.4) and by performing freeze–thaw cycles in dry ice/ethanol bath. The crude lysates were purified first by ultracentrifugation (1.5 h at 350,000 × *g* at 18 °C) in a discontinuous iodixanol gradient and the virus containing fractions were purified with ion-exchange chromatography using FPLC. Genome copy titers were determined using real time quantitative PCR^3^. Injected vector titer was 7.7 × 10^14^ genome copies/ml (gc/ml) for both rAAV2/6-hu-α-syn and rAAV2/6-GFP vectors.

### Surgery

Mice were anesthetized with a mixture of tiletamine/zolazepam (25 mg/kg) and xylazine (5 mg/kg) and placed on a stereotaxic apparatus (Stoelting, USA) with mouse adaptor and lateral ear bars. The head skin was cut longitudinally and vector solution was bilaterally injected within the dorsal hippocampus or the substantia nigra *pars compacta* (SNpc) using a 0.2 mm-gauge stainless steel injector connected to a 5 μl Hamilton syringe. In all experimental groups, the rAAV was injected in a volume of 1 μl/side for the SNpc and 0.5 μl/side for the dorsal hippocampus at a rate of 0.2 μl/min. For the SNpc, the stereotaxic coordinates used (flat skull position) were: antero-posterior (AP) = −3.1 mm, medio-lateral (ML) = ±1.3 mm, dorso-ventral (DV) = −4.5 mm and AP = −3.1 mm, ML = ± 0.6 mm, DV = −4.3 mm; for dorsal hippocampus: AP = −1.8 mm, ML = ±1.4 mm, DV = −1.6 mm, with AP and ML relative to bregma and DV from the dura, according to the mouse brain atlas^52^. Only animals with correct injection placements, verified by analyzing immunofluorescence staining of consecutive coronal brain sections, were included in the statistical analysis.

### Behavioral assessment

To study the early and long-term consequences due to α-Syn accumulation in the brain, animals were subjected to behavioral assessment after 4 weeks (short-term experiment) and 24 weeks (long-term experiment) after the initial vector injection. For the hippocampus experiments, mice were subjected to a battery of behavioral tasks, evaluating memory capacity in the 6-different objects task (6-DOT) and 6-identical objects task (6-IOT), spontaneous locomotor activity in the open field task, anxiety in the open field and elevated plus-maze tasks, sensorimotor gating in the startle-box, and associative learning in the contextual fear conditioning test. We report here all the behavioral data (in addition to those already published of the rotarod test) that were obtained from the animal that received bilateral injections of the same vectors analyzed in Giordano et al.^13^ Additionally, they were subjected to motor tests, including the rotarod test, which depends on the proper functioning of the nigro-striatal neurocircuitry^13^. After completion of the behavioral sessions, as previously done for the brains of animals injected in the SNpc/VTA^13^, the brains of hippocampal-injected mice were collected and processed for *post-mortem* analysis. Expression of hu-α-Syn and GFP animals was assessed in each animal by immunofluorescence analysis. Subgroups of these animals (as indicated in the figure legends) were used for biochemical and immunohistochemical experiments.

### Six different and six identical objects tasks

We tested object short-term memory capacity in mice by using the 6-DOT/6-IOT, which modifies the memory load by exposing the animals to six different (6-DOT, high load) and six-identical (6-IOT, low load) objects during the study phase. The original version of the task is used in a previous work^53^ and consisted of exposing the animals to 3, 4, 6, and 9 different objects (DOT) during the study phase, with the rationale that increasing the number of different objects increases the memory load. Each of these conditions was paired with its control task involving the same number of samples, but using identical objects (IOT), thus with low memory load. Based on previous evidence showing that hippocampal lesions selectively impair the 6-DOT, but not all the other tasks^53^, in this study we subjected the animals to the 6-DOT as a high memory load condition and to the 6-IOT as a low memory load control condition.

In both tasks, mice were left free to explore an empty arena (60 × 47 × 35 cm) during a 10 min habituation trial. The distance traveled (m) was recorded and used as an index of spontaneous locomotor activity. The first 5 min of this phase were used to assess the anxiety-like behavior of the animal, by measuring the time spent in the center of the arena, also defined as thigmotaxis^54^. During the study phase, animals were allowed to explore each different object for 35 s, meaning that they were allowed to collect a total of 35 s and 210 s in the 6-IOT and 6-DOT, in 5 min or 10 min maximum duration time, respectively. The different set of objects used were chosen based on pilot experiments that allowed to avoid basal differences for any of the sample objects and was identical to that used in previous studies^23,24,53,55^. After an inter-trial interval of 1 min, mice were exposed for 5 min to identical copies of the objects of the study phase, of which one was replaced by a novel object (test trial). Mice were exposed first to the 6-DOT and one week later to the 6-IOT, using different sets of objects. The exploration time, defined as the time the nose of active animals ranged within 2 cm from the object, was measured across all the different trials (study phase and test trial) of the 6-DOT and 6-IOT with Any-Maze (Ugo Basile, Varese, Italy) and was used to calculate the percentage preference for the novel object as follows: [(Novel object exploration during test trial/Total object exploration during test trial) × 100].

### Elevated plus maze

We used the elevated plus maze to measure anxiety-related behavior^56^. The maze (Ugo Basile, Varese, Italy) consists of four arms (two open without walls and two enclosed by 15 cm high walls) 35 cm long and 5 cm wide. Each arm of the maze was attached to sturdy metal legs such that it was elevated 60 cm from the ground. We used a digital video camera mounted overhead on the ceiling and Any-Maze as a video-tracking system to assess the time spent in the center, open and closed arms of the maze. We analyzed the percentage of time spent in the open arms according to the equation (1) (percentage of time spent in the open arms = time in the open arms/(time in the open + time in the closed arms) × 100) and we used it as an index of anxiety-like behavior.

### Acoustic startle response and pre-pulse inhibition test

The acoustic startle response (ASR) and pre-pulse inhibition (PPI) tests were used to assess stress reactivity and sensorimotor gating functions. The tests were performed with a startle reflex measuring apparatus (SR-LAB; San Diego Instruments, San Diego, CA), following a protocol already used^57^. The system comprises a piezoelectric unit that transduces vibrations into signals when mice startle inside a plexiglass cylinder. First, mice were placed in a plexiglass cylinder with background noise (65 decibel [db]) for 5 min (acclimation phase). Then, mice were subjected to a total of 42 trials, including: (a) startle trials (40 milliseconds [ms] with 80, 90, 100, 110 and 120 db acoustic pulses), (b) pre-pulse + startle trials: 20 ms with acoustic pre-pulses of 3 (68), 6 (71) and 12 (77) db above background noise followed, 100 ms later, by a 40 ms 120-db startling pulse. Startle amplitude was measured every 1 ms over a 65 ms period beginning at the onset of the startle stimulus. Average startle amplitude over the sampling period was taken as the dependent variable. Percent PPI at each pre-pulse intensity was calculated as [100−(startle response for pre-pulse/startle response for startle-alone trials) × 100].

### Fear conditioning

Mice were tested in a conditioning chamber (Ugo Basile, Varese, Italy). The chambers contained an infrared camera equipped with a speaker and located within a sound insulated box. The context contained a metal grid floor that was connected to an electric shock generator. This context contained no decorations and was cleaned with ethanol (EtOH) 30% between each subject. On day 1, mice were placed in context A for 6 min. Mice were allowed 180 s to explore the context before receiving any stimuli. Mice were then presented with a total of 3 foot shocks (0.5 mA, 2 s) each spaced at 60 s intervals. Following presentation of the final stimulus, mice remained in the context for 60 s. Afterwards, subjects were removed from the context and returned to their home-cage. On day 2, mice were exposed to the context for 5 min, without any added stimuli. Freezing behavior was assessed as the time (s) of absence of movements except for respiratory-related ones.

### Immunofluorescence procedure

Mice were perfused with 35–40 ml of 0.01 M phosphate buffer solution (1x PBS), followed by the same volume of 4% paraformaldehyde in 1x PBS (PFA). Four to six free-floating hippocampal or midbrain alternated coronal sections 30-μm thick were washed three times for 10 min with 1x PBS. Before washing in 1x PBS, slices for Proteinase K treatment were incubated with Proteinase K (Sigma Aldrich) at a concentration of 50 μg/mL for 2 min at room temperature (RT). Subsequently, all slices were incubated with a blocking solution (0.3% Triton TM X-100 and 5% normal goat serum (NGS) in 1x PBS) for 1 h. Slices were incubated overnight with primary antibodies against hu-α-Syn (1:1000, sc-12767, Santa Cruz Biotechnology) in combination with hu-α-Syn phosphorylated at Ser129 (phospho- S129-hu-α-Syn, 1:100, ab51253, Abcam) or NeuN (1:300, ABN90, Merck Millipore) or TH (1:1000, AB152, Millipore) or D1DR (1:200, sc-33660, Santa Cruz Biotechnology). Brain slices were incubated with the proper mixture of secondary antibodies: goat anti-rabbit Alexa Fluor® 647, goat anti-mouse Alexa Fluor® 488, goat anti-guinea pig Alexa-Fluor® 568). DAPI was used for nuclear counterstaining (1:1000, 10 min, D1306, Invitrogen) before slice mounting and inclusion with a Mowiol® 4-88 (Sigma Aldrich) solution. Representative mosaic slice reconstructions showing the spread of rAAV2/6-hu-α-Syn injection in the midbrain and the striatum (Fig. 2b) were acquired with a confocal microscope (Leica DM5500) with 20x magnification. Representative mosaic slice reconstructions showing the spread of rAAV2/6-hu-α-Syn and rAAV-GFP injections in the hippocampus, stained with hu-α-Syn and p-α-Syn (Fig. 4b) were acquired at 20x magnification with a Leica Laser Microdissector. p-α-Syn positive cells in the CA1 of the hippocampus were acquired with a confocal microscope (Leica DM5500) with 40x magnification (Fig. 4c). Images of TH immunostaining in the hippocampus and the striatum (Supplementary Fig. 1a, c) were acquired with a confocal microscope (Leica DM5500) at ×20 and ×10 magnification, respectively. Images for D1DR in the striatum and the hippocampus (Supplementary Fig. 1b) were acquired with a confocal microscope (Olympus, IX83) at 60x magnification. Images of NeuN and hu-α-Syn in the CA1, CA3 and DG of the hippocampus were acquired with a confocal microscope (Zeiss, LSM700 Axio Observer) at 40x magnification (Supplementary Fig. 3a). All images were acquired with a 1024×1024 pixel resolution. For all the experiments, at least 3-5 slices *per* mouse and 3-4 mice *per* group were acquired and analyzed. p-α-Syn positive cells (Fig. 4c) were counted manually after applying a threshold (the same for all conditions) in the pyramidal layer of the CA1 of each hemisphere, using a macro for the cell counter plugin of FIJI (ImageJ, NIH, USA); data from the two hemispheres were averaged *per* slice and the final cell count was averaged *per* mouse. TH immunoreactivity in the CA1 of the hippocampus and the striatum was detected as the mean intensity value in regions of interest (ROIs) with the use of FIJI; data were expressed as percentage of r-AAV-GFP mice. The number of D1DR spots were identified and automatically measured in the CA1 of the hippocampus using QuPath 0.4.3^58^; each image was sampled with 6 ROIs (1300 µm^2^), and spots were automatically counted with a fixed detection threshold and settings of spot size as follows: min spot size 0.1 µm^2^, expected spot size 1 µm^2^ and maximum spot size 2 µm^2^; data were averaged *per* slice and expressed as percentage of r-AAV-GFP mice. The number of NeuN^+^ neurons per slice was determined in the CA1, CA3 and DG of the hippocampus with the use of FIJI; data were then summed per CA1, CA3 and DG of each slice, averaged per slice of each experimental condition, and normalized per group of hippocampal antero-posterior coordinates. In the same slices the area of hu-α-Syn spread was quantified (mm^2^) from the whole hippocampus images with the use of FIJI.

### Sample preparation for western blotting and dot-blot

Briefly, sample preparation, western blotting and dot-blot, were performed through dissection of dorsal hippocampi by punching the area surrounding the dorsal CA1 sectioned with the use of a mouse brain blocker (1 mm, Kopf Instruments); the SNpc/VTA was manually dissected by separating it from the surrounding areas. Samples were homogenized in RIPA buffer (50 mM Tris-HCl pH 7.4, 1% Triton X-100, 0.5% Na-deoxycholate, 0.1% SDS, 150 mM NaCl, 2 mM EDTA and protease/phosphatase inhibitor cocktail). After protein quantification (Bradford, BioRad), 10 µg of protein were loaded on an SDS-polyacrylamide gel and transferred to PVDF membranes. Membranes were incubated overnight at 4 °C with the following primary antibodies: GluA1 (1:500; ab31232; Abcam), GluA2 (1:500; ab20673; Abcam), p-s845 (1:500; 36-8300; Invitrogen), p-s831 (1:500; AB5847; Millipore), LC3 (1:3000; NB100-2220; Novus Bio), p62 (1:500; H00008878-M01; Tebu-Bio), Synapsin 1/2 (1:1000; 106002; Synaptic System), SNAP-25 (1:1000; 111002; Synaptic System), Snapin (1:1000; 148002; Synaptic System), VAMP2 (1:1000; 104202; Synaptic System); Synaptophysin (1:1000, ab8049, Abcam), vGluT1 (1:1000; 135304; Synaptic System), CSPα (1:1000, AB1576, Millipore), PSD-95 (1:1000, 124011, Synaptic System), D1DR (1:1000, sc-33660, Santa Cruz), Tyrosine Hydroxylase (1:1000, AB152, Abcam), NMDAR1 (1:1000, 320500, Invitrogen), NMDAR2A (1:1000, M264, Sigma) and NMDAR2B (1:1000, 718600, Invitrogen). β-actin (1:5000, MAB1501, Millipore) was used as loading control. The appropriate secondary antibody was incubated (1:5000, BioRad) for 1 h at room temperature. Immunoreactivity was detected by chemiluminescence and bands were quantified by densitometry using ImageJ software (NIH). We reported data normalized to the GFP group because we calculated the percentage relative to the control for each blot, to correct for any type of differences relative to different gels (for example recycling of primary antibody or time to ECL exposure)^59^.

For the dot-blot of hu-α-Syn, 150 ng of proteins were spotted on a nitrocellulose membrane 0.22 μm thick. The membrane was boiled for 15 min in phosphate buffered saline (PBS), blocked with 5% non-fat dry milk and probed with primary antibody against hu-α-Syn (1:100; sc-12767; Santa Cruz Biotechnology) overnight at 4 °C^60^. For pan-α-Syn detection, 150 ng of protein were spotted on a nitrocellulose membrane 0.22 μm thick. The membrane was blocked with 5% non-fat dry milk and probed with primary antibody (1:1000; sc-69977; Santa Cruz Biotechnology) overnight at 4 °C. After incubation with the appropriate secondary antibody, immunoreactivity was detected by chemiluminescence and spots were quantified by densitometry using ImageJ software. Ponceau S staining was used as loading control. We reported optical density normalized on Ponceau staining.

All blots were processed in parallel and derive from the same experiment.

### Electrophysiological extracellular field potential

For electrophysiological recordings, male mice (*n* = 8 for both experimental groups) were anesthetized with 2-bromo-2-chloro-1,1,1 -trifluoroethane and then sacrificed. The brain was then rapidly removed, dissected, and immersed in ice-cold Krebs’ solution, artificial cerebrospinal fluid (ACSF) containing (126 mM NaCl, 2.5 mM KCl, 1.2 mM MgCl_2_, 1.2 mM NaH_2_PO_4_, 2.4 mM CaCl_2_, 10 mM glucose, and 25 mM NaHCO_3_) oxygenated with a carbogen mixture gas (CO_2_ 5% and O_2_ 95%). Hippocampal (400 μm thick) slices were cut using a vibratome and were allowed to recover in ACSF continuously bubbled with carbogen at room temperature for 1–2 h before experimental recordings. Each slice was transferred in a registration chamber, and under visual control, by means of a stereoscope, a stimulating electrode was inserted into the Schaffer collateral fibers and a recording electrode was inserted into the CA1 region (Fig. 5f)^6^. Field excitatory postsynaptic potentials (fEPSPs) of half-maximal amplitude were evoked, with a single electric pulse, every 10 s; I/O curves were obtained from an average of the slope of 5 fEPSPs at the same stimulation steps (every 4 mV) and normalized from 0 to 100% taking the minimum and the maximum response; LTP was induced by a high-frequency stimulation (HFS) protocol constituted by one train at 100 Hz.

### Statistical analysis

Results are expressed as mean ± standard error (S.E.M.). Statistical analyses were performed using StatView 5.0 (SAS Institute Inc., North Carolina, USA), SigmaPlot 11.0 (Systat Software, Chicago, IL, USA), InStat 3.0 (GraphPad, San Diego, CA, USA) and Statistica 10.0 (Statsoft, Tulsa, OK, USA). Statistical significance was assessed using two-tailed paired or unpaired *t* test, Mann–Whitney test, ANOVA or repeated measured ANOVA, followed by the Duncan post-hoc comparison test when appropriate. For single object comparisons, we used repeated measures with objects (six levels: New, F1-F5), followed by Dunnett post hoc analysis. Before applying the ANOVA test, data distribution normality was tested with the Kolmogorov–Smirnov test. For electrophysiology, T-test and Two-way ANOVA were used for statistical analysis. Significance was set at P < 0.05. The number of animals used for each experiment, the degrees of freedom and the number of recorded cells used for statistical analysis are reported in the figure legends. Some animals were excluded from statistical analysis in case of procedural problems, such as lost videos.

### Reporting summary

Further information on research design is available in the Nature Research Reporting Summary linked to this article.

## Supplementary information

Supplementary Materials_Iemolo et al

Reporting Summary

## Data Availability

The data that support the findings of this study are available from the corresponding author upon request.
