# Supplementary material for: Synaptic mechanisms underlying onset and progression of memory deficits caused by hippocampal and midbrain synucleinopathy
Source: NPJ Parkinsons Dis. 2023 Jun 16;9:92. doi: 10.1038/s41531-023-00520-1 (PMC10275870; doi:10.1038/s41531-023-00520-1)
Supplement: Supplementary file 1 — Supplementary Materials_Iemolo et al [file 41531_2023_520_MOESM1_ESM.pdf]

Supplementary Information for

Synaptic mechanisms underlying onset and progression of memory deficits caused by hippocampal and midbrain synucleinopathy

Attilio Iemolo[1,2*], Maria De Risi[1,3*], Nadia Giordano[1#], Giulia Torromino[3,4#], Cristina Somma[1], Diletta Cavezza[3], Martina Colucci[1,2], Maria Mancini[5], Antonio de Iure[6], Rocco Granata[3], Barbara Picconi[6,7], Paolo Calabresi[8,9], Elvira De Leonibus[1,2,3§]

1. Telethon Institute of Genetics and Medicine, Via dei Campi Flegrei 34, Pozzuoli, Naples, Italy.
2. Institute of Genetics and Biophysics (IGB), Consiglio Nazionale delle Ricerche (CNR); via Pietro Castellino 111, Naples (Italy).
3. Institute of Biochemistry and Cell Biology, Consiglio Nazionale delle Ricerche (CNR), Via Ramarini 33, Monterotondo Scalo, Rome, Italy.
4. University of Naples Federico II, Department of Humanistic Studies, Italy.
5. Institute of Neuroscience (IN), Consiglio Nazionale delle Ricerche (CNR), via Raoul Follereau 3, Vedano al Lambro, Monza e Brianza, Italy.
6. Lab. Experimental Neurophysiology, IRCCS San Raffaele Roma, 00166, Italy.
7. Telematic University San Raffaele, Rome, 00166, Italy.
8. Neurological Clinic, Fondazione Policlinico Universitario Agostino Gemelli IRCCS, 00168 Rome, Italy.
9. Neurology, Department of Neuroscience, Faculty of Medicine, Università Cattolica del "Sacro Cuore", 00168 Rome, Italy.

* These authors contributed equally
\# These authors contributed equally

Email address: elvira.deleonibus@cnr.it

This PDF file includes:

- Supplementary Figures 1 to 6
- Supplementary Table 1
- Uncropped dot/western blots

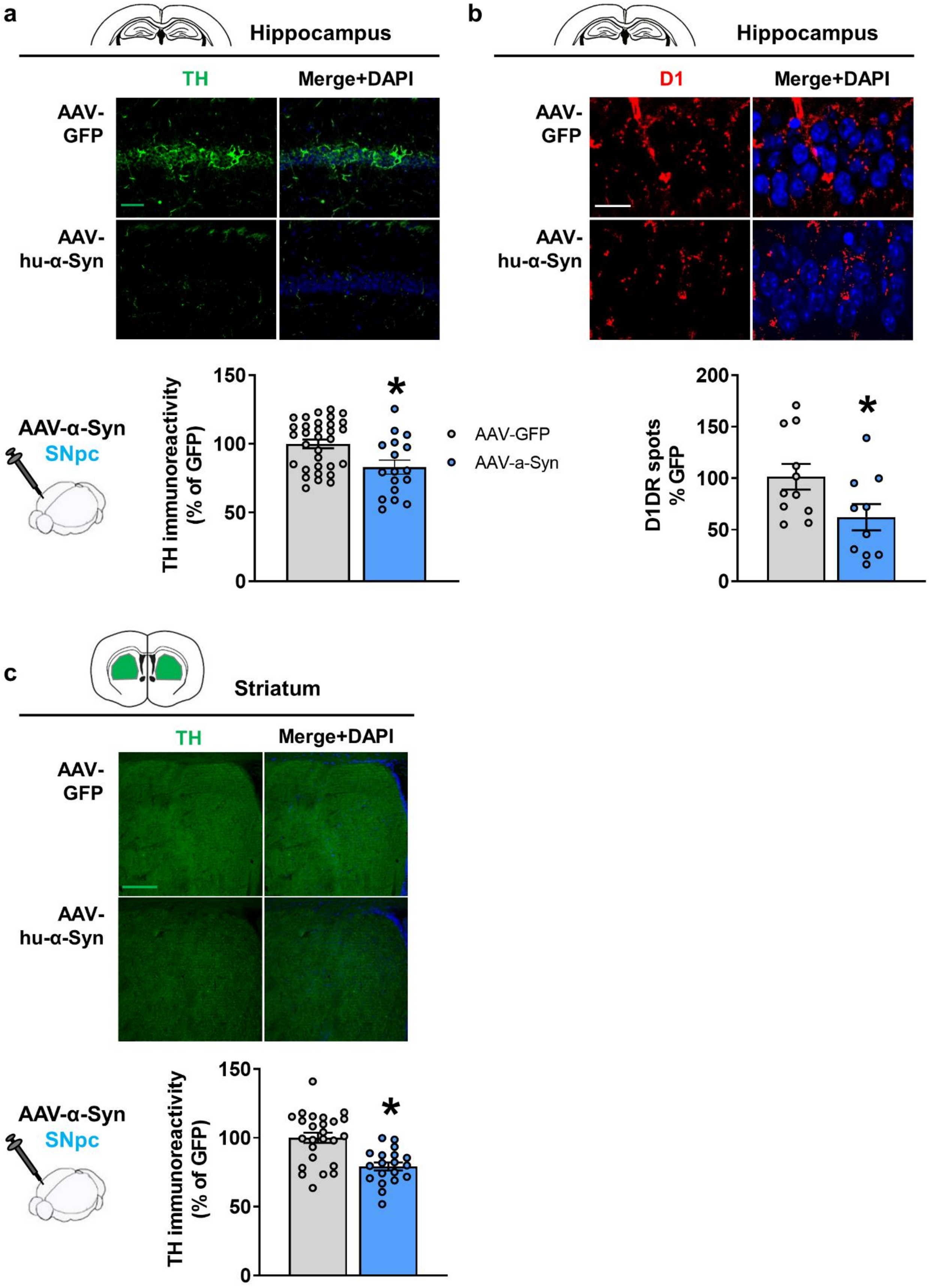

a
Hippocampus
TH
Merge+DAPI
AAV-GFP
AAV-hu-α-Syn
AAV-α-Syn
SNpc
TH immunoreactivity (% of GFP)
150
100
50
0
*
AAV-GFP
AAV-a-Syn
b
Hippocampus
D1
Merge+DAPI
AAV-GFP
AAV-hu-α-Syn
D1DR spots % GFP
200
150
100
50
0
*
c
Striatum
TH
Merge+DAPI
AAV-GFP
AAV-hu-α-Syn
AAV-α-Syn
SNpc
TH immunoreactivity (% of GFP)
150
100
50
0
*

Supplementary Figure 1. rAAV-mediated overexpression of hu-α-Syn in the midbrain leads to reduced TH immunoreactivity in the striatum and the hippocampus and decreases the number of D1R1 spots in the latter. (a) Overexpression of hu-α-Syn in the SNpc/VTA significantly decreased the percentage of TH immunoreactivity compared to SNpc-GFP mice in the hippocampus (unpaired t test, $t = 2.973$, $P = 0.0046$, SNpc-GFP, $n = 32$; SNpc-hu-α-Syn, $n = 17$). Representative confocal images of the CA1 are reported for each condition in the upper panel (Scale bar: 50 μm). **(b)** In parallel, SNpc-hu-α-mice show a reduction of D1DR spots compared to rAAV-GFP mice (unpaired t test, $t = 2.194$, $P = 0.0409$, SNpc-GFP, $n = 11$; SNpc-hu-α-Syn, $n = 10$). Representative confocal images of D1DR spots are reported for each condition in the upper panel (Scale bar: 20 μm). **(c)** SNpc-hu-α-mice show a significantly decreased percentage of TH immunoreactivity in the striatum compared to SNpc-GFP mice (unpaired t test, $t = 4.215$, $P < 0.0001$, SNpc-GFP, $n = 25$; SNpc-hu-α-Syn, $n = 19$). Representative confocal images of the striatum are reported for each condition in the upper panel (Scale bar: 200 μm). Data are presented as mean ± SEM. * $P < 0.05$ from SNpc-GFP.

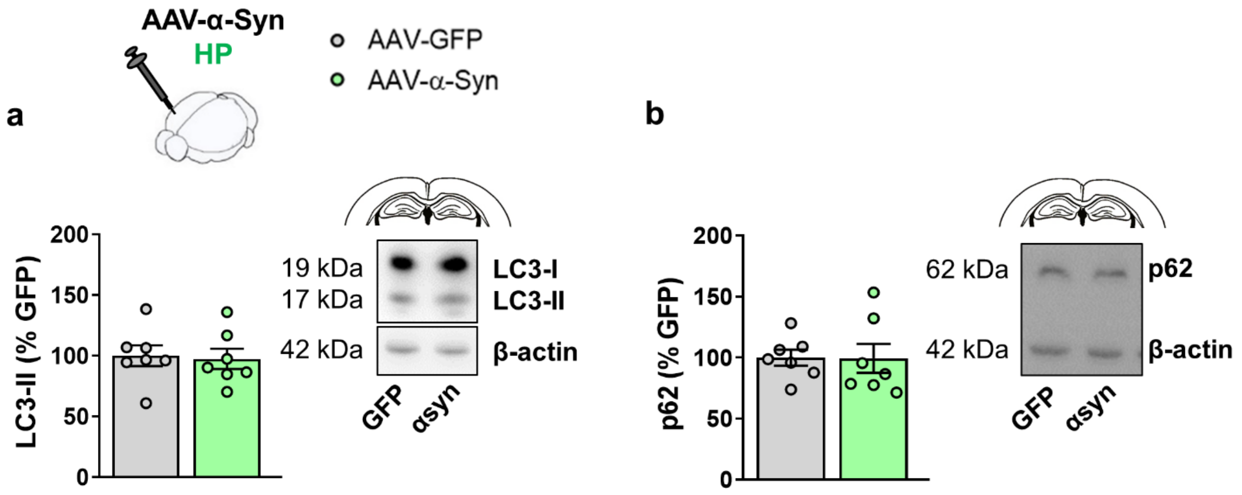

Supplementary Figure 2. rAAV-mediated overexpression of hu-α-Syn does not lead to autophagy impairment. (**a**) Western blot analysis of the autophagosomal membrane marker LC3-II and (**b**) the autophagy substrate p62 in the hippocampus of HP-hu-α-Syn and HP-GFP mice. Representative bands of S2 belong to the same blot of GluA2 reported in Fig. 5d. Data are presented as mean ± SEM.

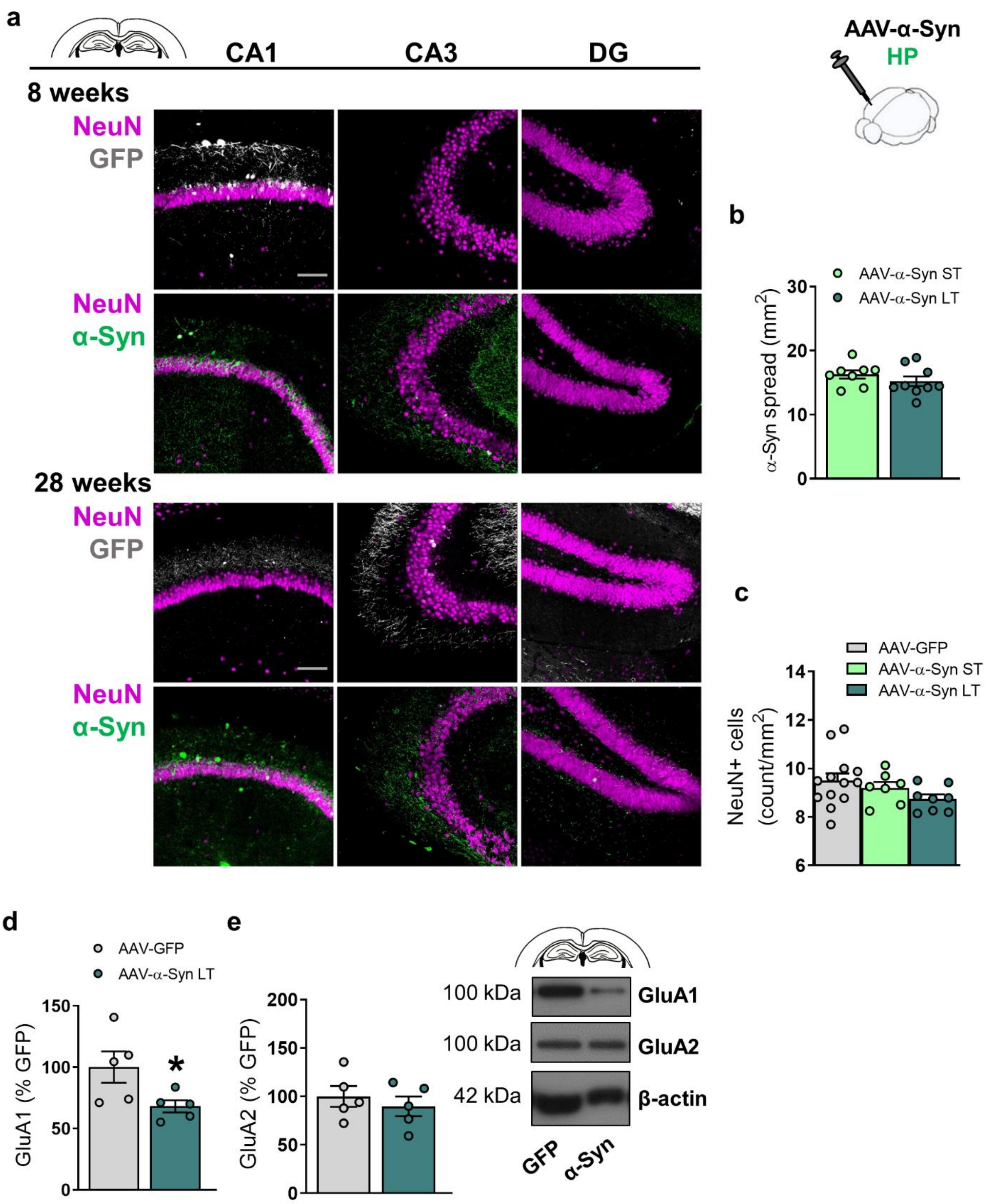

Supplementary Figure 3. Short and long-term rAAV-mediated overexpression of GFP and hu-α-Syn in the hippocampus does not lead to neuronal loss. (**a**) Representative confocal images from CA1, CA3 and DG of control GFP (grey) and hu-α-Syn (green) mice, stained for NeuN (magenta) in the dorsal hippocampus (Scale bar: 100 µm). (**b**) Quantification of hu-α-Syn spread in the dorsal hippocampus after short- and long-term overexpression showed no significant difference between

groups. (**c**) Accumulation of α-Syn in the dorsal hippocampus after short- and long-term overexpression of hu-α-Syn does not induce a significant reduction in NeuN-immunoreactivity, when compared to rAAV-GFP control animals. (**d**) Long-term overexpression of hu-α-Syn in the hippocampus decreased GluA1 (unpaired t test, $t = 2.329$, $P = 0.04$, HP-GFP, $n = 5$; α-Syn, $n = 5$) but not GluA2 (**e**), expression. Representative bands for each condition are reported. Data are presented as mean ± SEM. * $P < 0.05$ different from rAAV-GFP.

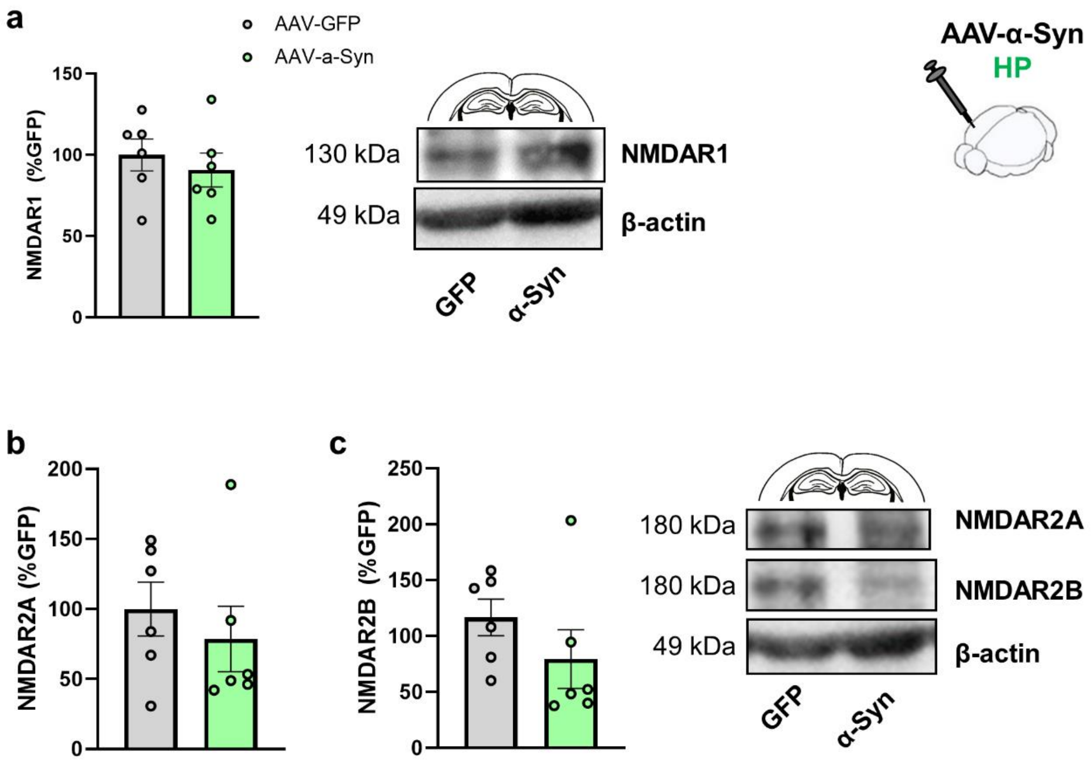

Supplementary Figure 4. Short-term rAAV-mediated overexpression of hu-α-Syn in the hippocampus does not affect the expression of NMDA receptor subunits. (**a-c**). Western blot analysis of the hippocampal tissue in the HP-hu-α-Syn and HP-GFP groups for the NMDAR1 (**a**), NMDAR2A (**b**) and NMDAR2B (**c**) subunits (n = 6 for each experimental group). Representative bands for each condition are reported. Data are presented as mean ± SEM.

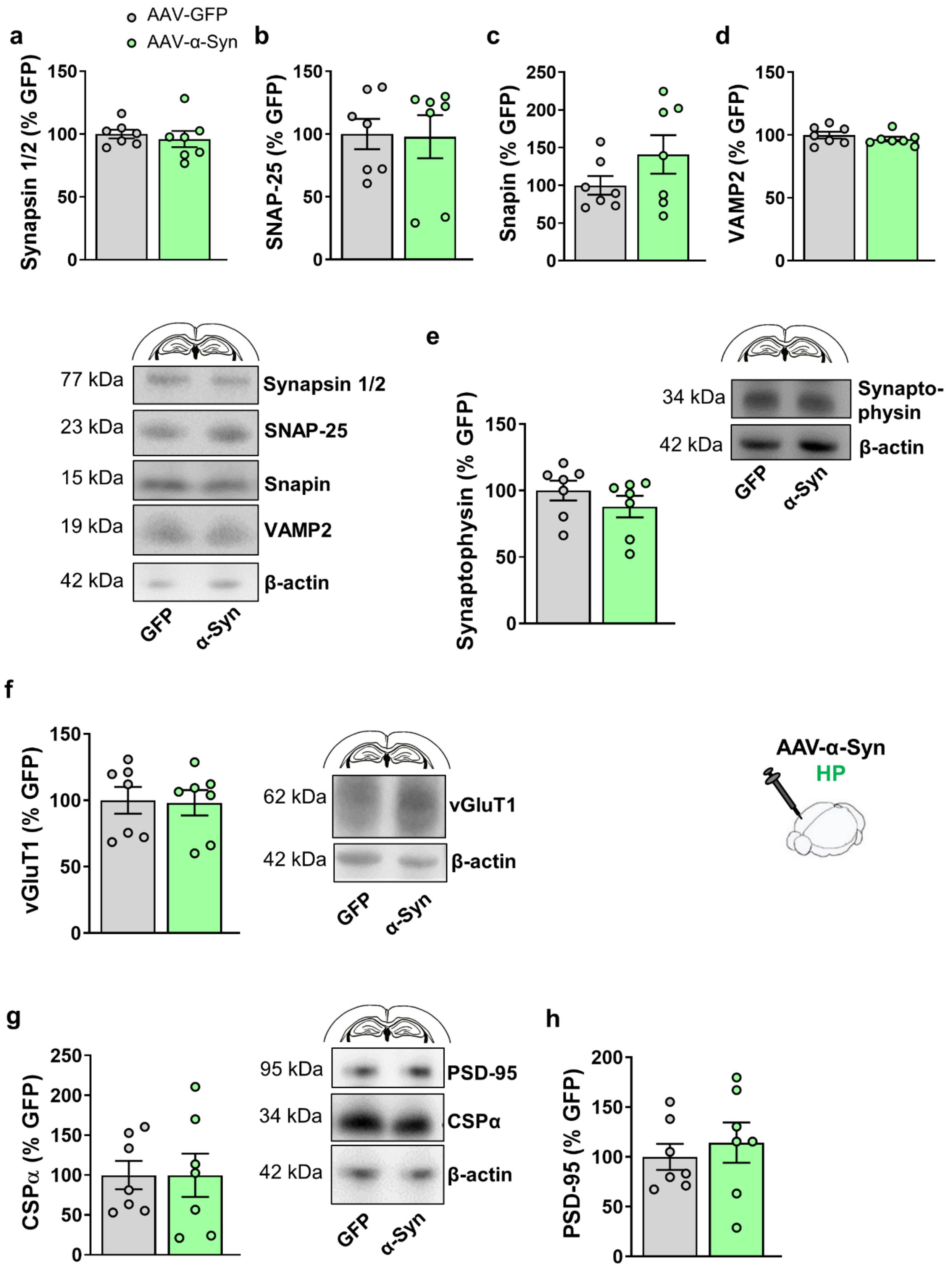
a
b
c
d
e
f
g
h
AAV-GFP
AAV-α-Syn
Synapsin 1/2 (% GFP)
SNAP-25 (% GFP)
Snapin (% GFP)
VAMP2 (% GFP)
77 kDa
23 kDa
15 kDa
19 kDa
42 kDa
Synapsin 1/2
SNAP-25
Snapin
VAMP2
β-actin
GFP
α-Syn
Synaptophysin (% GFP)
34 kDa
Synapto-physin
vGluT1 (% GFP)
62 kDa
vGluT1
AAV-α-Syn
HP
CSPα (% GFP)
95 kDa
PSD-95
CSPα
PSD-95 (% GFP)

Supplementary Figure 5. Short-term rAAV mediated overexpression of hu-α-Syn in the hippocampus does not affect the expression of pre- and post-synaptic density proteins. (**a-h**) Short-term accumulation of hu-α-Syn in the mouse hippocampus does not change the expression of several essential proteins involved in synapse function and morphology (GFP, n = 7; hu-α-Syn, n = 7). Representative bands for each condition are reported. Data are presented as mean ± SEM.

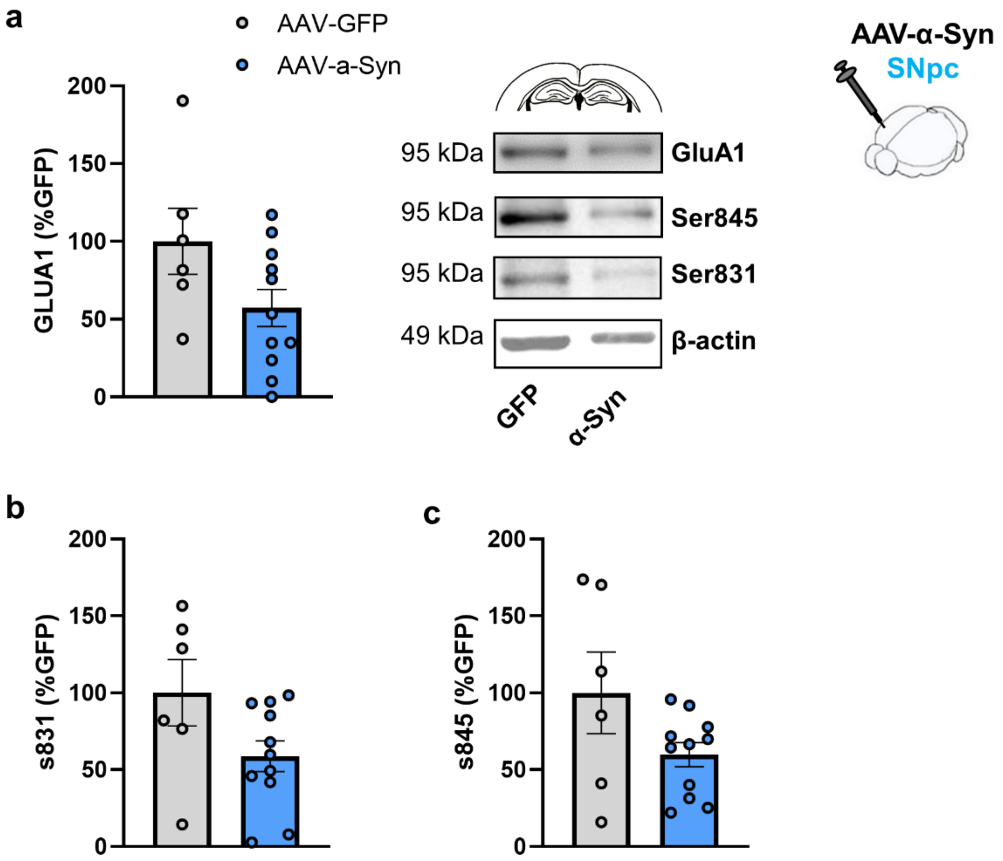

Supplementary Figure 6. Long-term overexpression of hu-α-Syn in the midbrain does not affect the hippocampal expression of GluA1 AMPA receptors and phosphorylation. (**a-c**) Western blot analysis of the hippocampal tissue from SNpc-hu-α-Syn mice showing no significant change in the expression of GluA1 and its phosphorylation at serine 845-831 sites compared to SNpc-GFP control (GFP, n = 6; hu-α-Syn, n = 11). Representative bands for each condition are reported. Data are presented as mean ± SEM.

Supplementary Table 1. Number of mice used for behavioral analysis.

	HIPPOCAMPUS				SUBSTANTIA NIGRA			
Behavior	4 weeks		24 weeks		4 weeks		24 weeks	
	HP-GFP	HP-hu-α-Syn	HP-GFP	HP-hu-α-Syn	SNpc-GFP	SNpc-hu-α-Syn	SNpc-GFP	SNpc-hu-α-Syn
6-DOT	8	11 (2)	9	15	15 (1)	19 (1)	8 (1)	15
6-IOT	8	13	9	15	15 (1)	20	8 (1)	15
Locomotor activity	7[1]	13	9	15	16	20	9	15
Open field (time in center)	7 [1]	13	9	15	16	20	9	15
Elevated plus maze	8	13	9	15	16	20	9	15
Pre-pulse inhibition	7 [1]	11 [2]	9	15	16	20	9	15
Fear conditioning	8	13	9	15	**ND**	**ND**	**ND**	**ND**
The number of excluded animals because of procedural testing problems or because they did not explore the objects for more than 5 sec is reported in square and round brackets, respectively.								

FIG 2C Dot blot anti-hu-αsyn; ponceau staining

DOT-BLOT#1

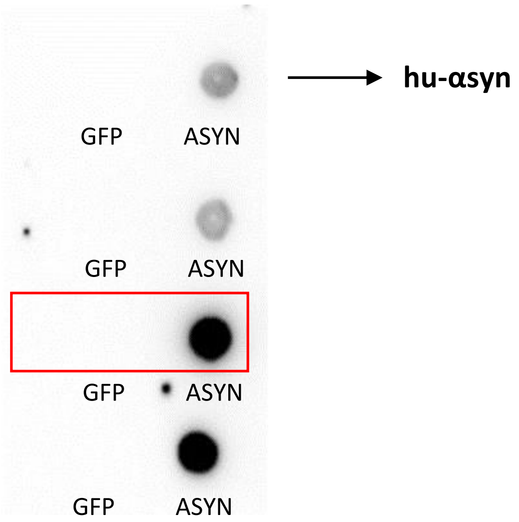

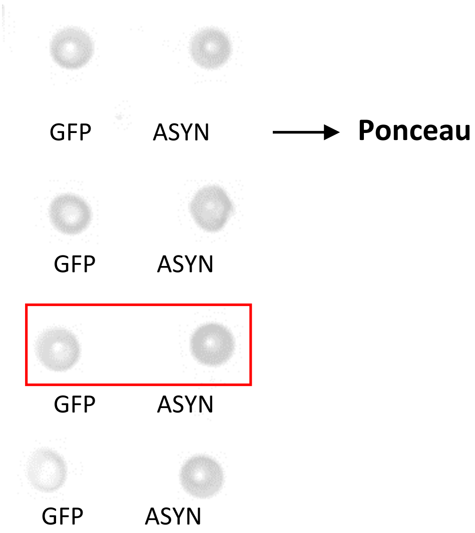

DOT-BLOT#2

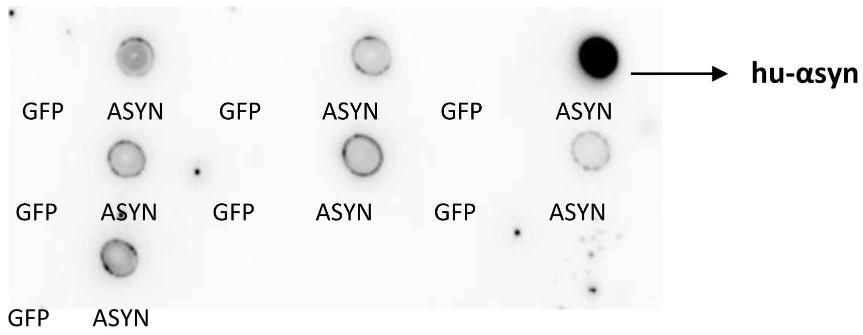

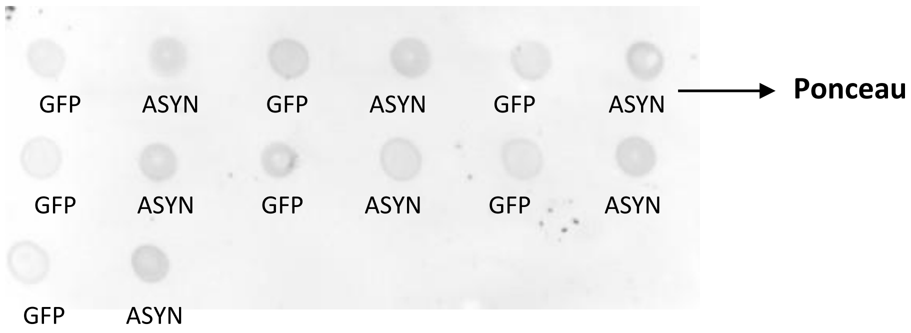

The square box represents bands reported in the figure

FIG 2D Dot blot anti-pan-αsyn; ponceau staining

DOT-BLOT#1

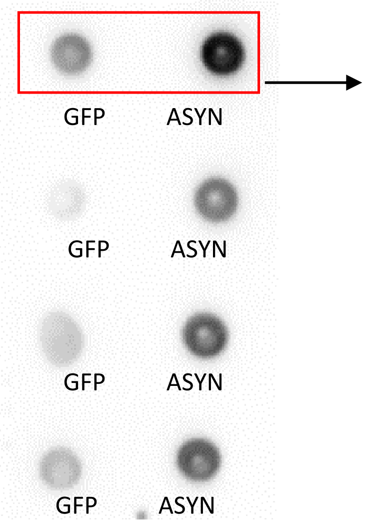

pan-αsyn

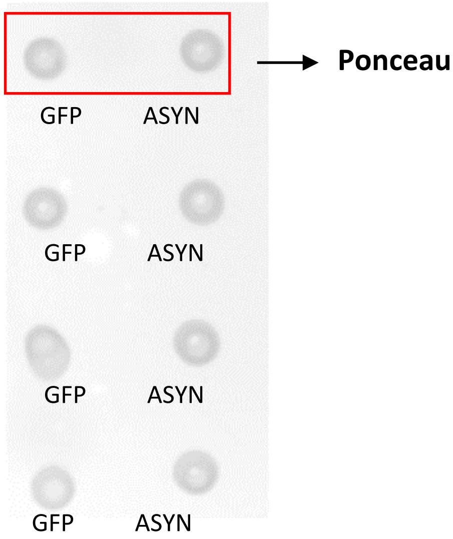

Ponceau

DOT-BLOT#2

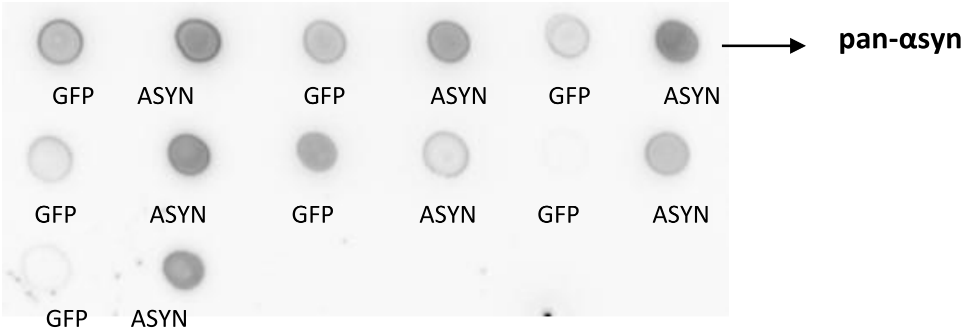

pan-αsyn

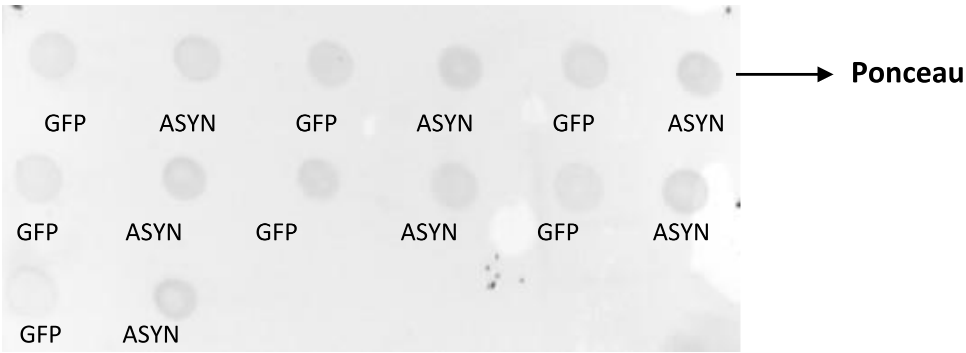

Ponceau

The square box represents bands reported in the figure

FIG 2E,F Anti TH, anti-D1R, anti-β-actin

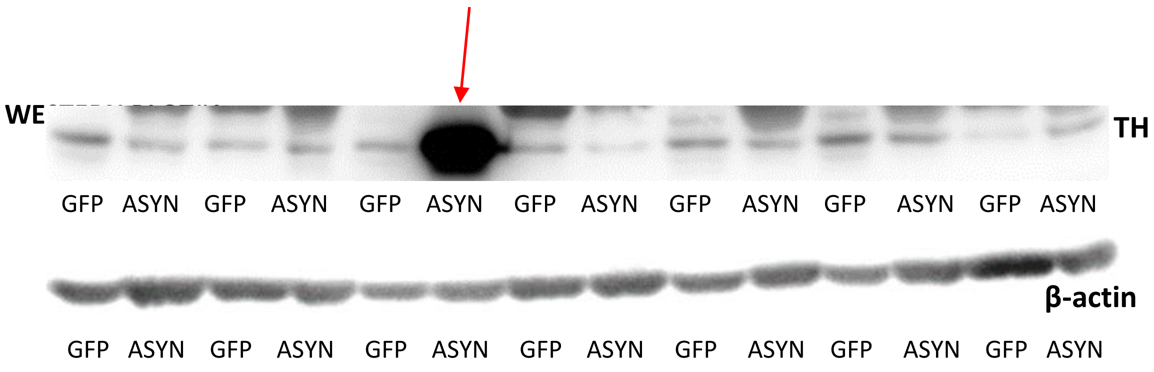

WESTERN BLOT#2

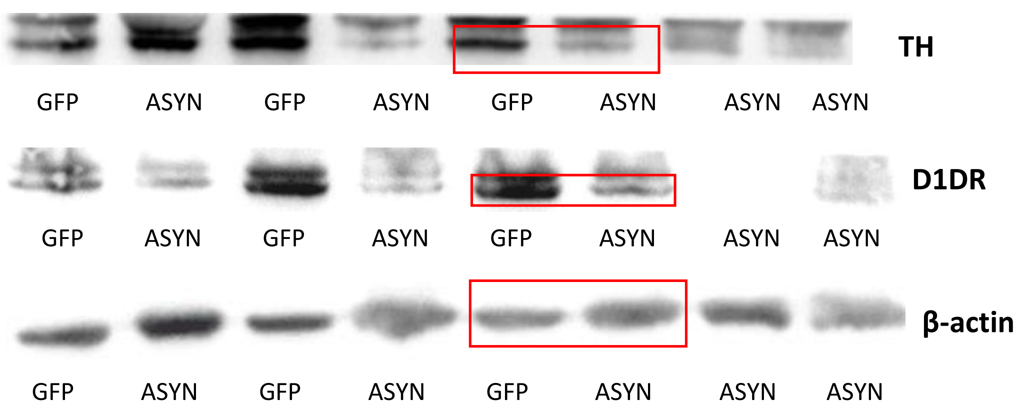

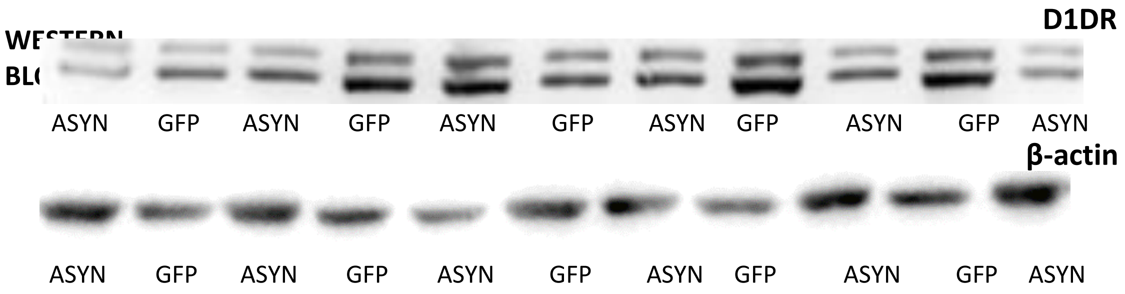

The arrow shows an outlier sample excluded from the analysis

The square box represents bands reported in the figure

Fig. 4d Dot blot anti-hu-αsyn; ponceau staining

DOT BLOT #1

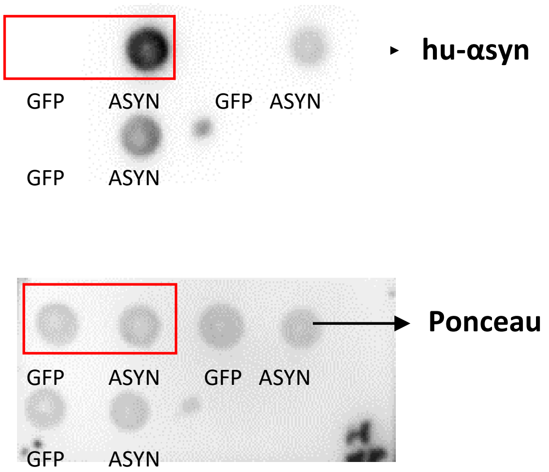

DOT BLOT #2

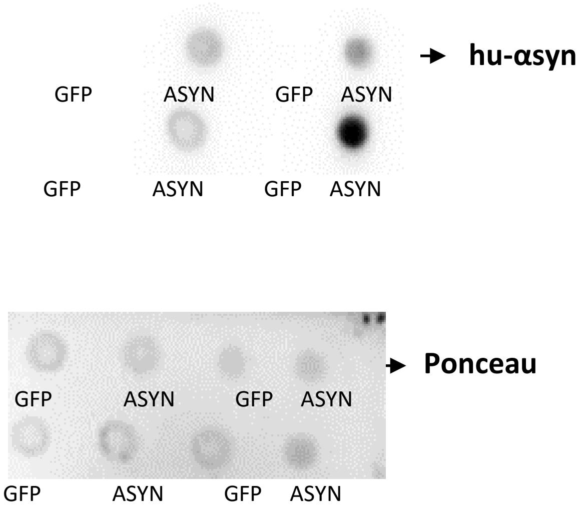

The square box represents bands reported in the figure

Fig. 4e Dot blot anti-pan-αsyn; ponceau staining

DOT BLOT #1

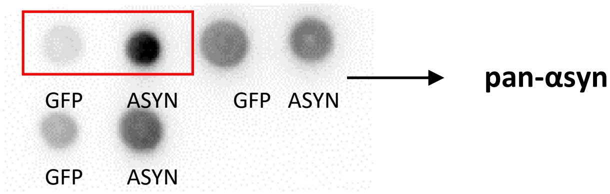

→ **pan-αsyn**

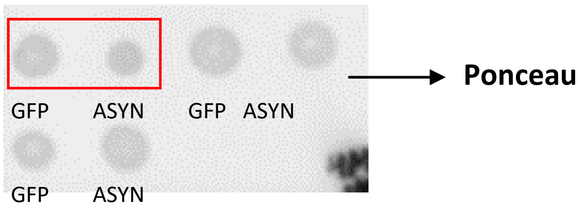

→ **Ponceau**

DOT BLOT #2

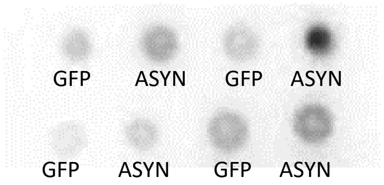

→ **pan-αsyn**

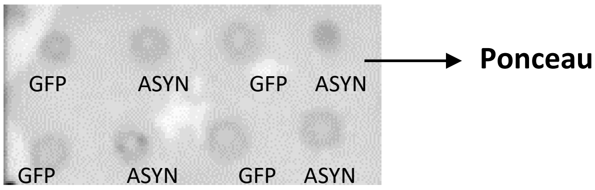

→ **Ponceau**

The square box represents bands reported in the figure

Fig. 5A, B, C anti- GluA1; anti-s845; anti-s831; anti-β-actin

WESTERN BLOT #1

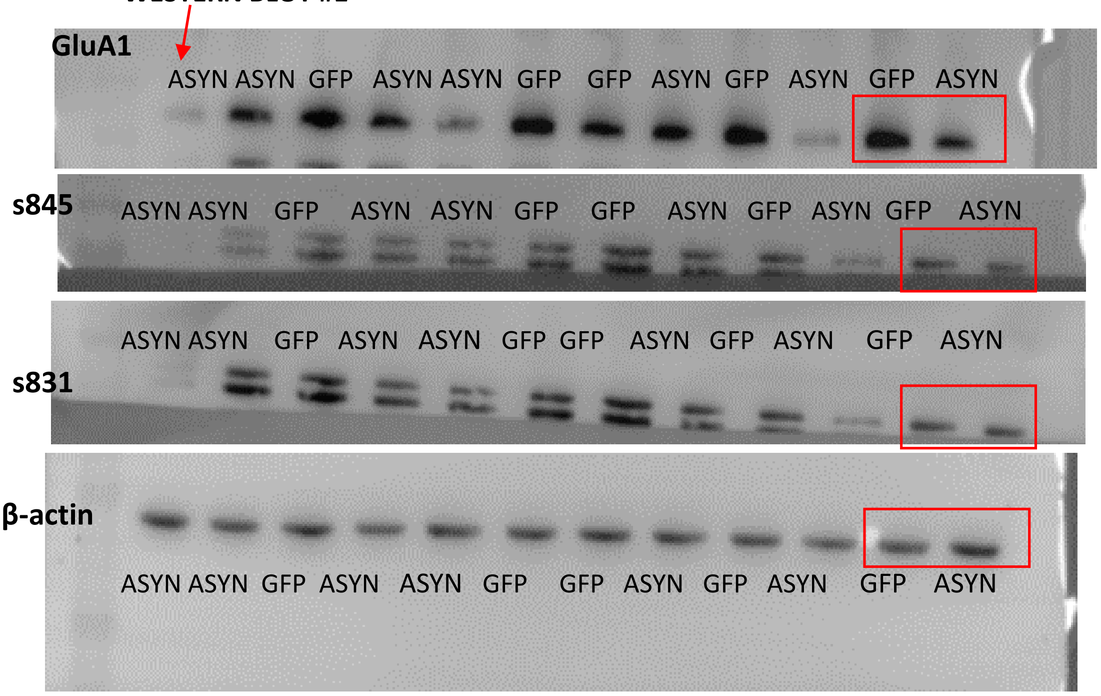

WESTERN BLOT #2

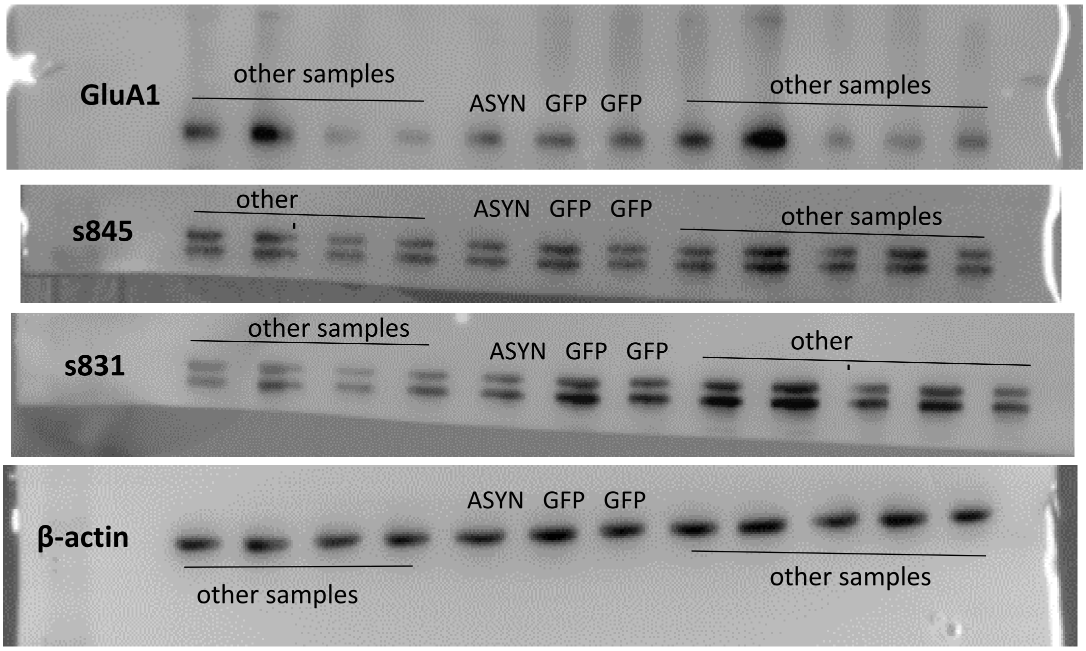

The square box represents bands reported in the figure
The first sample blot #1 was excluded and replicated in the second blot (arrow)

Fig. 5D anti- GluA2; anti-β-actin

WESTERN BLOT #1

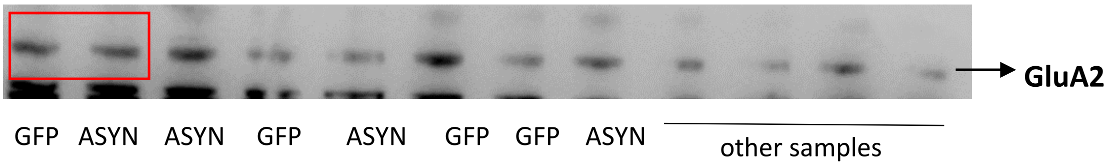

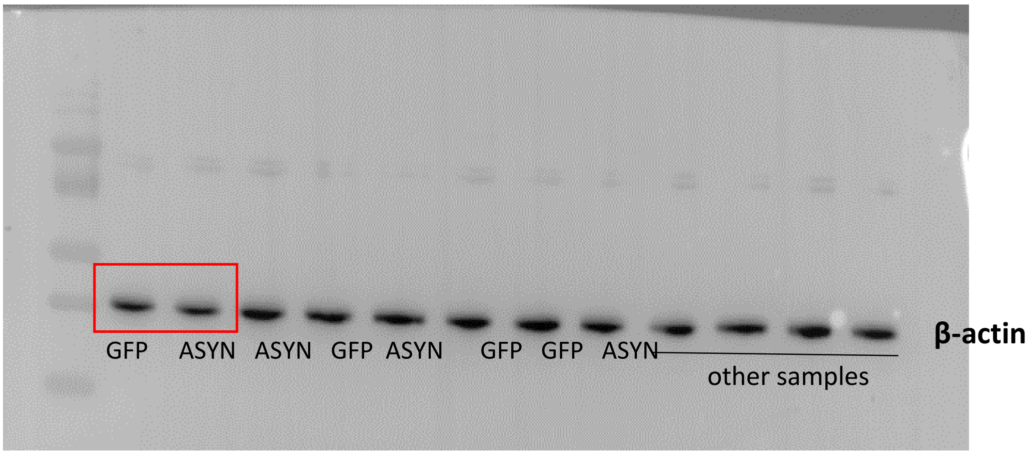

WESTERN BLOT #2

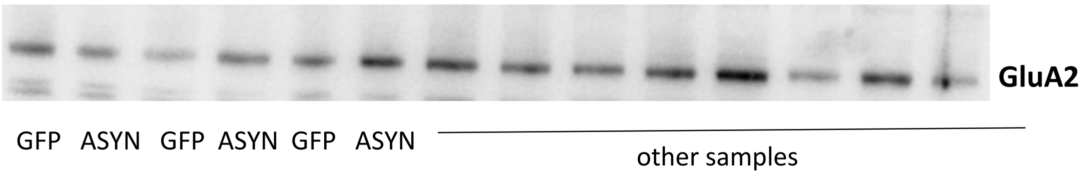

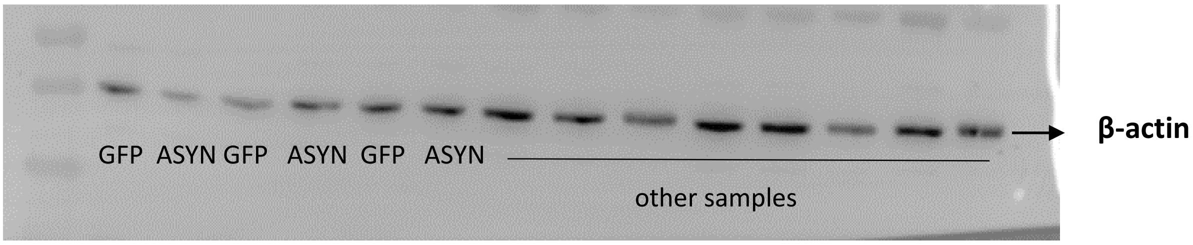

The square box represents bands reported in the figure
These are the same gel of figure S2a. GluA2 was blotted on the same membrane

Fig. S2a anti- LC3; anti-β-actin

WESTERN BLOT #1

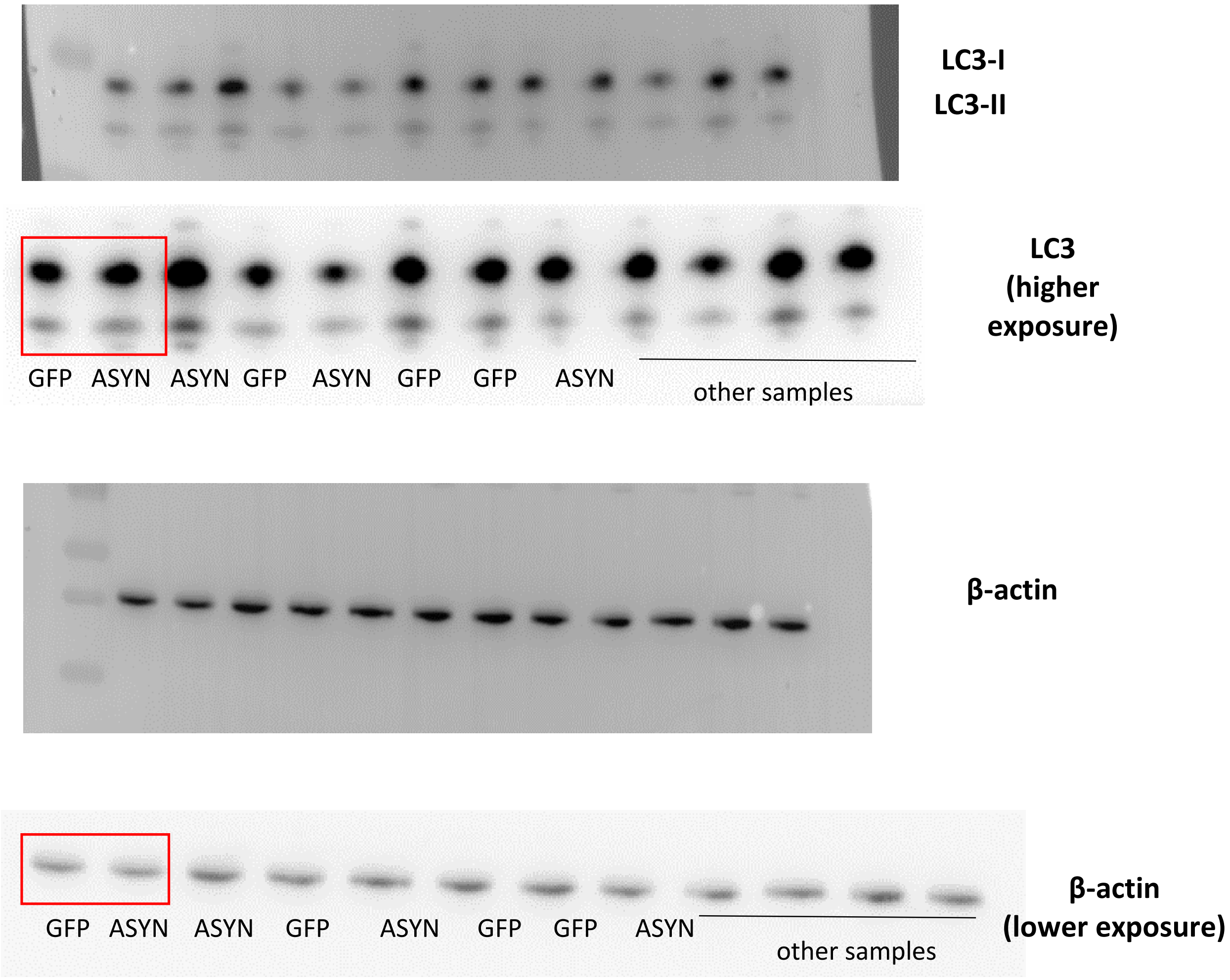

WESTERN BLOT #2

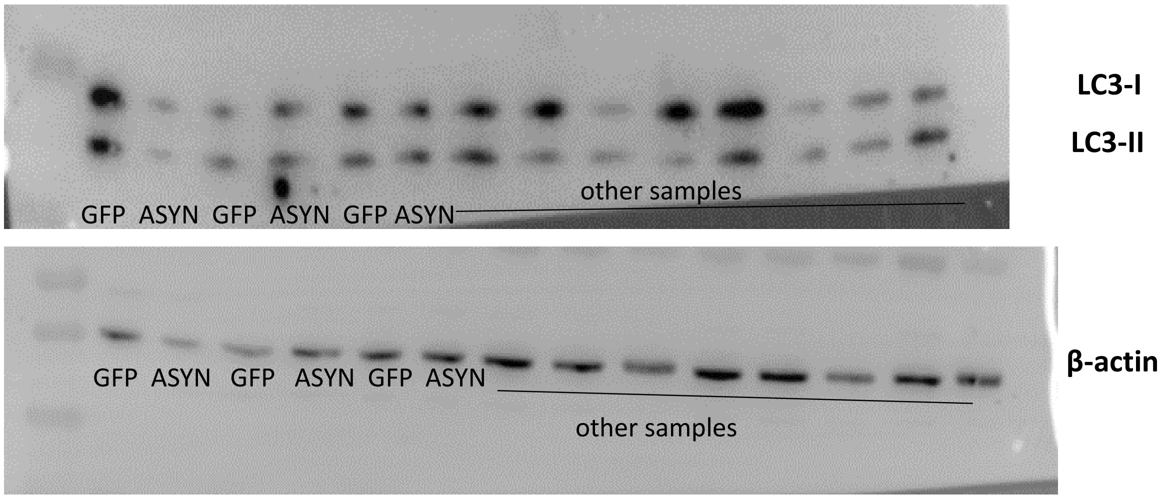

The square box represents bands reported in the figure
These are the same gel of figure 5D. LC3 was blotted on the same membrane

Fig. S2b anti- p62 anti-β-actin

WESTERN BLOT #1

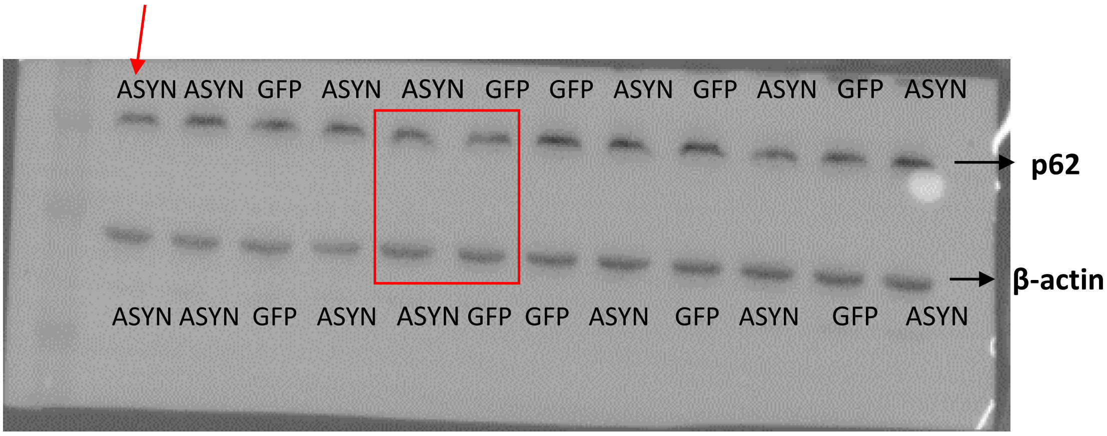

WESTERN BLOT #2

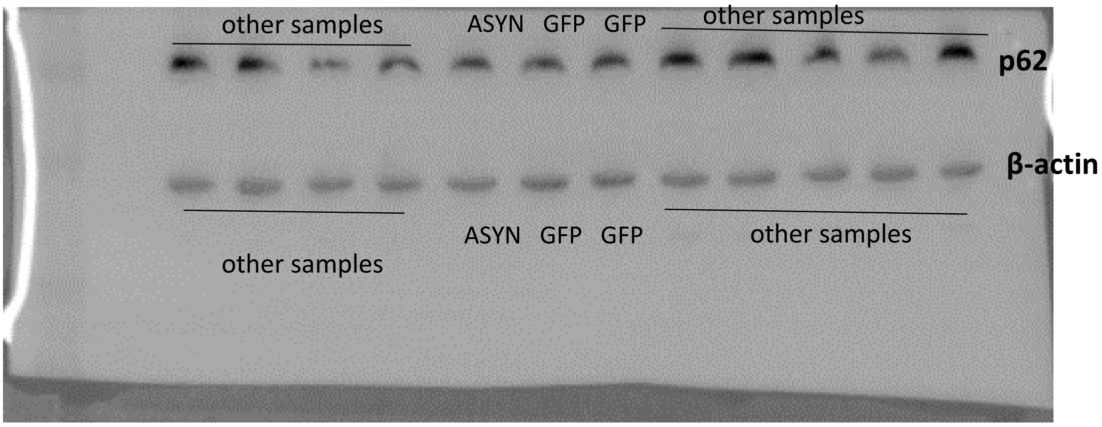

The square box represents bands reported in the figure
These are the same gel of figure 5A-C. P62 was blotted after the stripping
The first sample blot #1 was excluded and replicated in the second blot (arrow)

Fig. S3D-E anti- GluA1; anti- GluA2; anti-β-actin

WESTERN BLOT #1

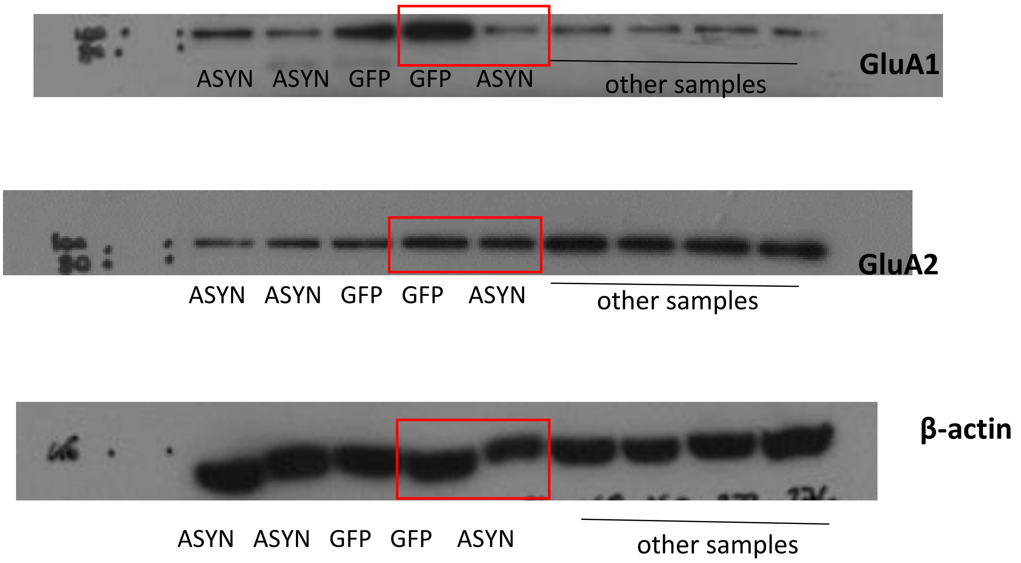

WESTERN BLOT #2

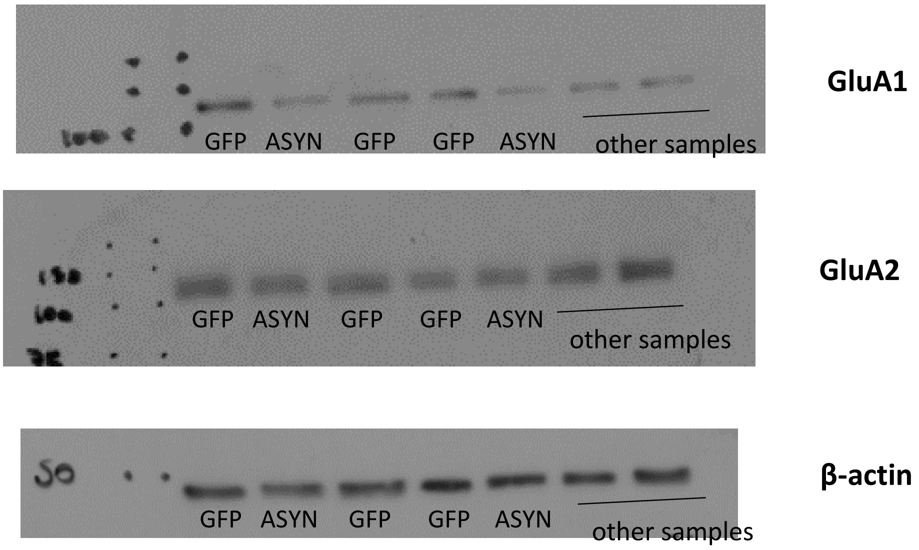

The square box rapresents bands reported in the figure

Fig S4A,B,C Anti NMDAR1, anti-NMDAR2A, anti-NMDAR2B, anti-β-actin

WESTERN BLOT#1

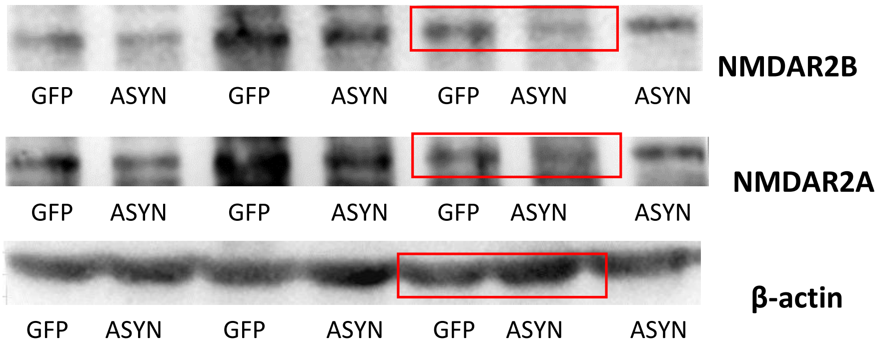

WESTERN BLOT#2

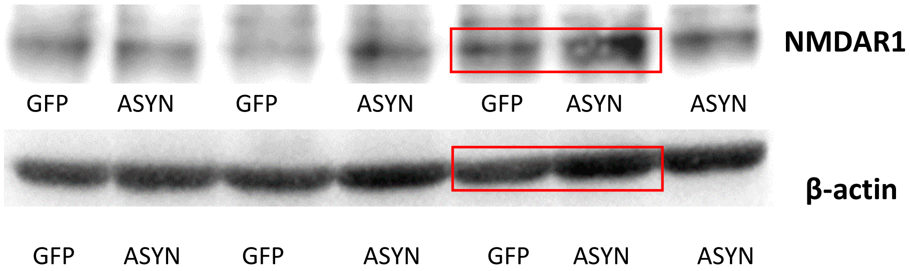

WESTERN BLOT#3

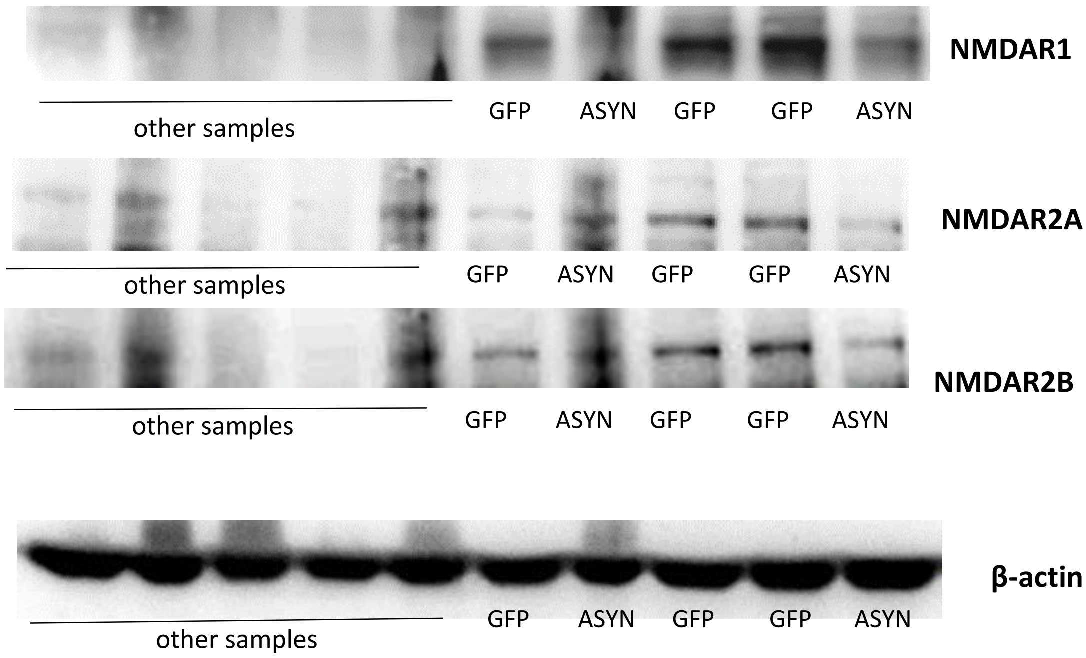

The square box rapresents bands reported in the figure

Fig. S5A, B, C, D Anti-Synapsin-1; anti-SNAP-25; anti-Snapin; anti-VAMP2; anti-β-actin

WESTERN BLOT #1

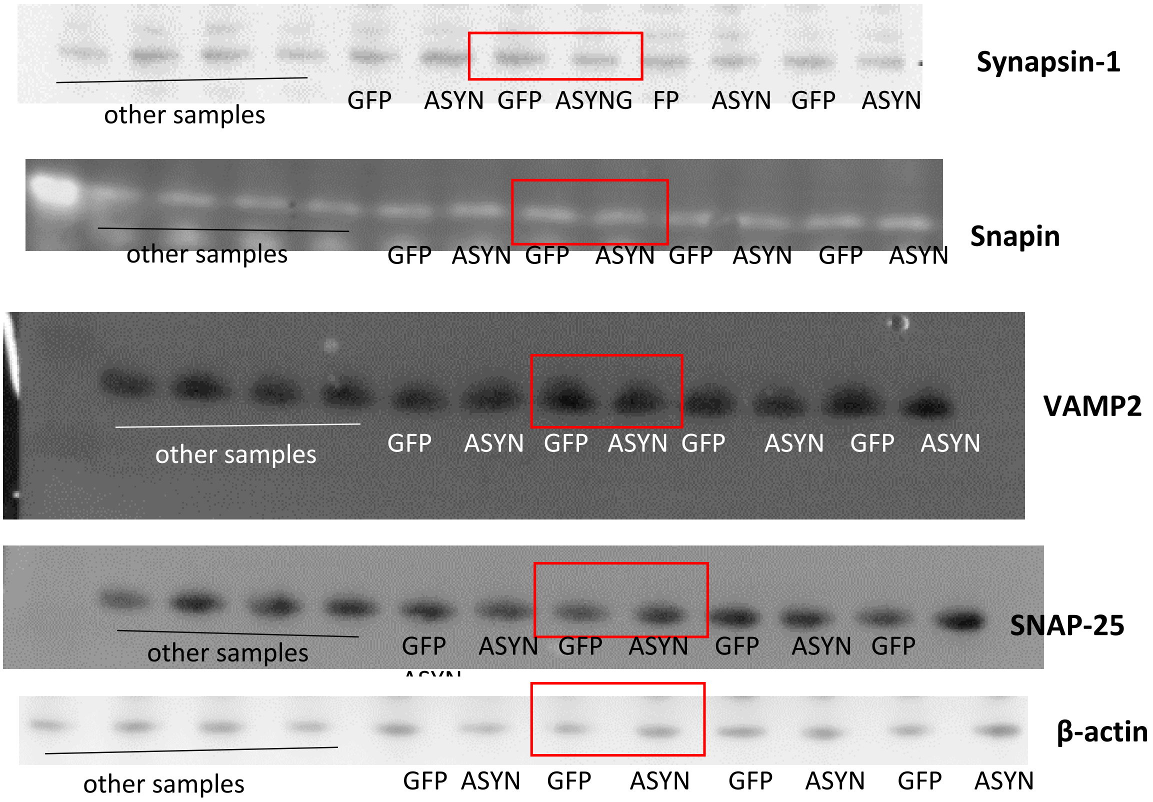

WESTERN BLOT #2

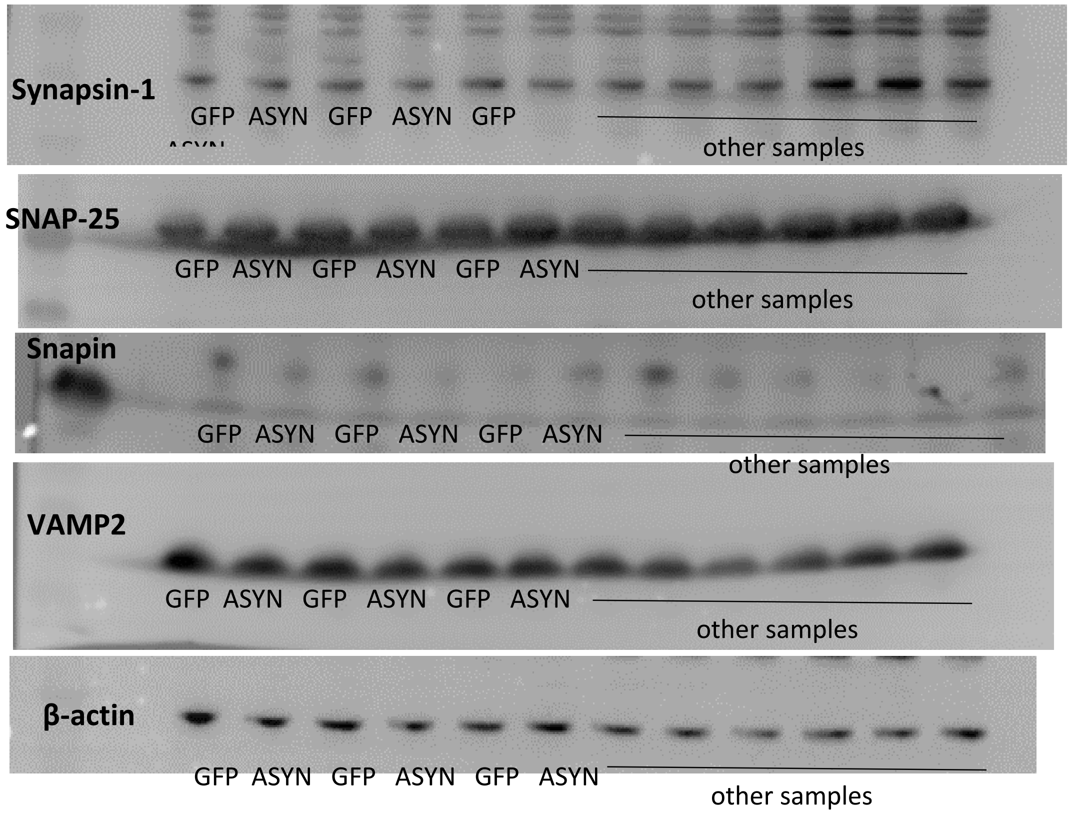

The square box represents bands reported in the figure
Fig. S5E Anti-Synaptophysin; anti-β-actin

WESTERN BLOT #1

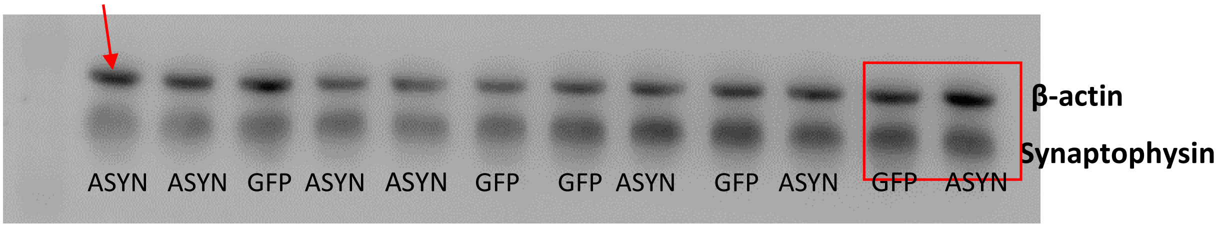

WESTERN BLOT #2

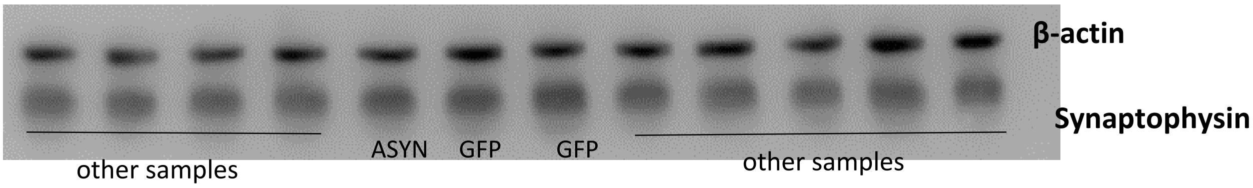

The square box rapresents bands reported in the figure
The first sample blot #1 was excluded and replicated in the second blot (arrow)
These are the same gel of figure 5A-C. Synaptophysin was blotted on the same membrane

Fig. S5F Anti-vGlut1; anti-β-actin

WESTERN BLOT #1

vGlut1

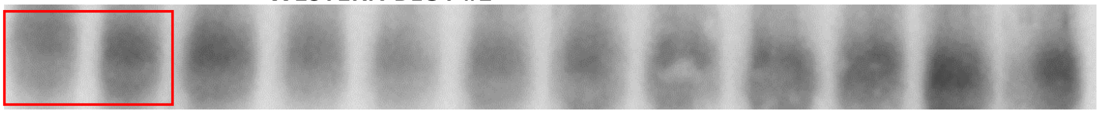

GFP ASYN GFP ASYN GFP ASYN GFP ASYN other samples

β-actin

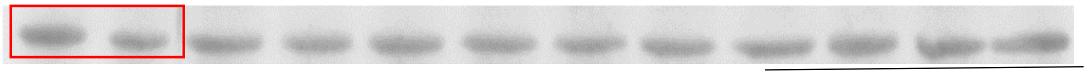

GFP ASYN GFP ASYN GFP ASYN GFP ASYN other samples

WESTERN B

vGlut1

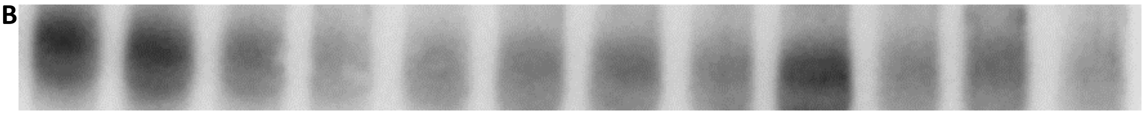

GFP ASYN GFP ASYN GFP ASYN other samples

β-actin

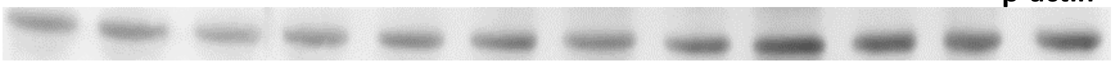

GFP ASYN GFP ASYN GFP ASYN other samples

The square box rapresents bands reported in the figure

Fig. S5G-H Anti-PSD-95; anti-CSP-alpha; anti-β-actin

WESTERN BLOT #1

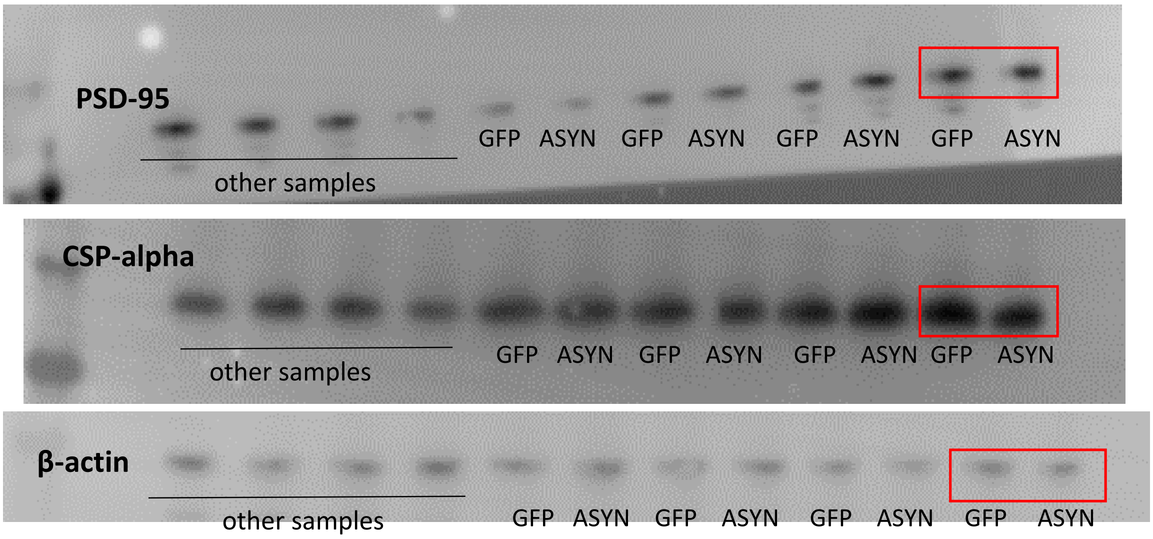

WESTERN BLOT #2

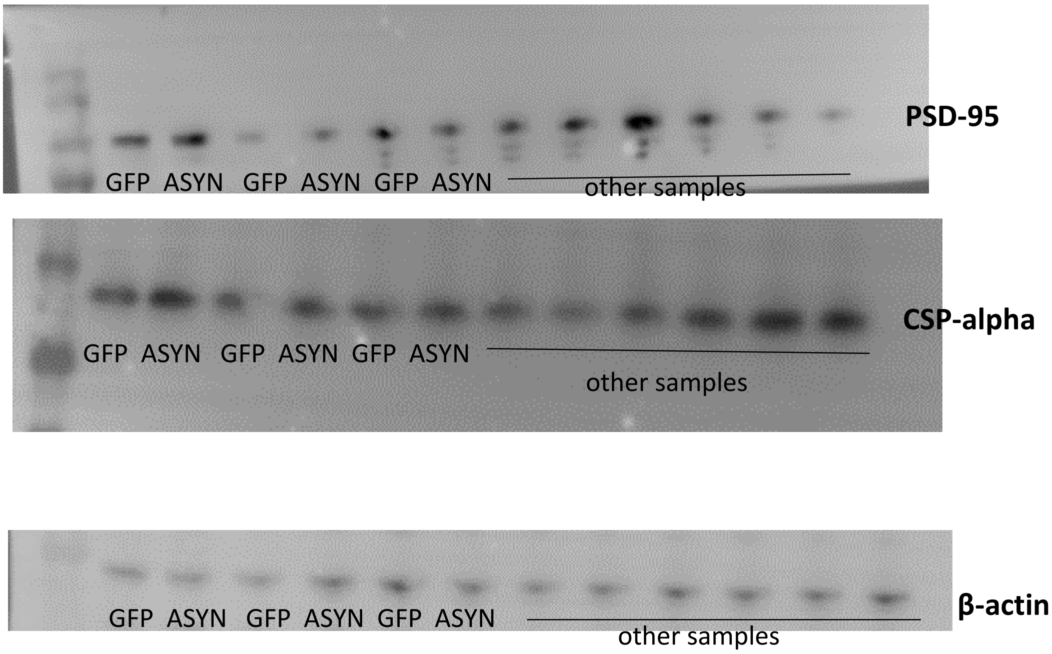

The square box rapresents bands reported in the figure

anti- GluA1; anti-s845; anti-s831; anti-β-actin

Fig S6 A,B,C

Western Blot #1

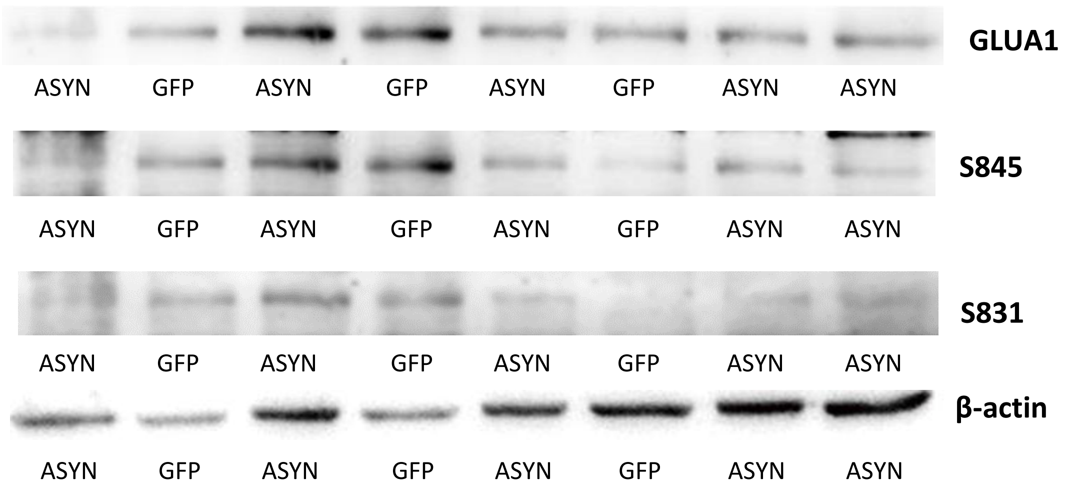

WESTERN BLOT#2

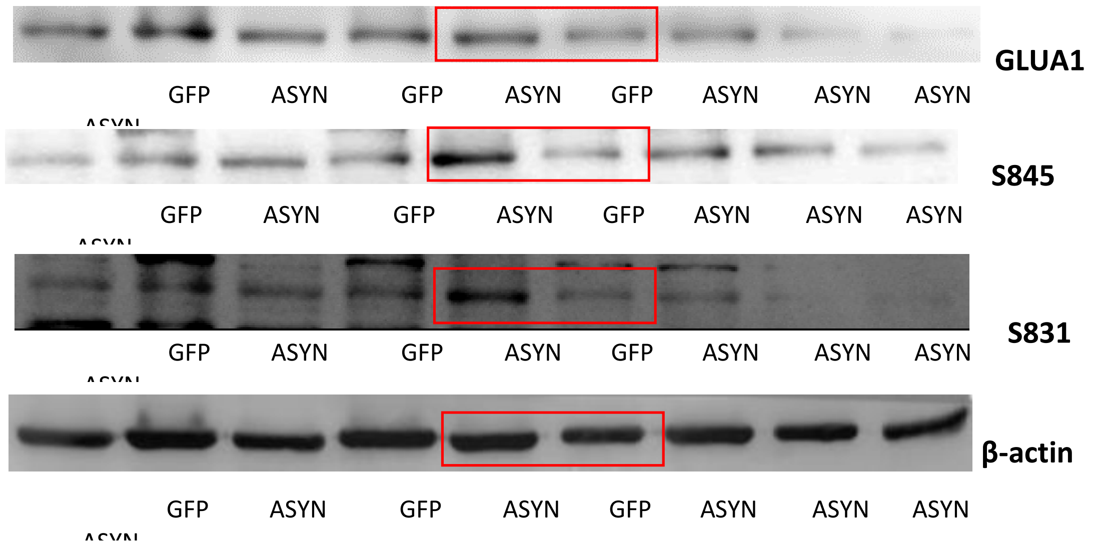

The square box rapresents bands reported in the figure
