# Supplementary material for: Synaptic mechanisms underlying onset and progression of memory deficits caused by hippocampal and midbrain synucleinopathy
Source: NPJ Parkinsons Dis. 2023 Jun 16;9:92. doi: 10.1038/s41531-023-00520-1 (PMC10275870; doi:10.1038/s41531-023-00520-1)
Supplement: Supplementary file 2 — Reporting Summary [file 41531_2023_520_MOESM2_ESM.pdf]

nature portfolio

Corresponding author(s): Elvira de Leonibus

Last updated by author(s): May 11, 2023

Reporting Summary

Nature Portfolio wishes to improve the reproducibility of the work that we publish. This form provides structure for consistency and transparency in reporting. For further information on Nature Portfolio policies, see our Editorial Policies and the Editorial Policy Checklist.

Statistics

For all statistical analyses, confirm that the following items are present in the figure legend, table legend, main text, or Methods section.

n/a	Confirmed	
☐	☒	The exact sample size (*n*) for each experimental group/condition, given as a discrete number and unit of measurement
☐	☒	A statement on whether measurements were taken from distinct samples or whether the same sample was measured repeatedly
☐	☒	The statistical test(s) used AND whether they are one- or two-sided<br>*Only common tests should be described solely by name; describe more complex techniques in the Methods section.*
☒	☐	A description of all covariates tested
☐	☒	A description of any assumptions or corrections, such as tests of normality and adjustment for multiple comparisons
☒	☐	A full description of the statistical parameters including central tendency (e.g. means) or other basic estimates (e.g. regression coefficient) AND variation (e.g. standard deviation) or associated estimates of uncertainty (e.g. confidence intervals)
☐	☒	For null hypothesis testing, the test statistic (e.g. *F*, *t*, *r*) with confidence intervals, effect sizes, degrees of freedom and *P* value noted<br>*Give P values as exact values whenever suitable.*
☒	☐	For Bayesian analysis, information on the choice of priors and Markov chain Monte Carlo settings
☒	☐	For hierarchical and complex designs, identification of the appropriate level for tests and full reporting of outcomes
☒	☐	Estimates of effect sizes (e.g. Cohen's *d*, Pearson's *r*), indicating how they were calculated

Our web collection on statistics for biologists contains articles on many of the points above.

Software and code

Policy information about availability of computer code

Data collection	Statview 5.0, SigmaPlot 11.0, InStat 3.0, Statistica 10.0 and Fiji ImageJ
Data analysis	Statview 5.0 and Statistica 10.0

For manuscripts utilizing custom algorithms or software that are central to the research but not yet described in published literature, software must be made available to editors and reviewers. We strongly encourage code deposition in a community repository (e.g. GitHub). See the Nature Portfolio guidelines for submitting code & software for further information.

Data

Policy information about availability of data

All manuscripts must include a data availability statement. This statement should provide the following information, where applicable:

- Accession codes, unique identifiers, or web links for publicly available datasets
- A description of any restrictions on data availability
- For clinical datasets or third party data, please ensure that the statement adheres to our policy

Data are available from the corresponding author on request. Source data underlying Figs. 2c-d, 2e-f, 4d-e, 5a-d and Supplementary Figs. 2a-b,3d-e, 4a-c, 5a-h, 6a-c are provided in Supplementary Materials file.

Research involving human participants, their data, or biological material

Policy information about studies with human participants or human data. See also policy information about sex, gender (identity/presentation), and sexual orientation and race, ethnicity and racism.

Reporting on sex and gender	N/A
Reporting on race, ethnicity, or other socially relevant groupings	N/A
Population characteristics	N/A
Recruitment	N/A
Ethics oversight	N/A

Note that full information on the approval of the study protocol must also be provided in the manuscript.

Field-specific reporting

Please select the one below that is the best fit for your research. If you are not sure, read the appropriate sections before making your selection.

☒ Life sciences ☐ Behavioural & social sciences ☐ Ecological, evolutionary & environmental sciences

For a reference copy of the document with all sections, see nature.com/documents/nr-reporting-summary-flat.pdf

Life sciences study design

All studies must disclose on these points even when the disclosure is negative.

Sample size	Sample size was chosen based on previous literature.
Data exclusions	Some animals were excluded from statistical analysis in case of procedural problems, such as lost videos
Replication	The reproducibility of the experimental findings was verified by replicating the same results using different cohorts of animals.
Randomization	Mice were allocated into experimental groups in random order.
Blinding	The investigators were blinded to experimental condition

Reporting for specific materials, systems and methods

We require information from authors about some types of materials, experimental systems and methods used in many studies. Here, indicate whether each material, system or method listed is relevant to your study. If you are not sure if a list item applies to your research, read the appropriate section before selecting a response.

Materials & experimental systems

n/a	Involved in the study	
☐	☒	Antibodies
☒	☐	Eukaryotic cell lines
☒	☐	Palaeontology and archaeology
☐	☒	Animals and other organisms
☒	☐	Clinical data
☒	☐	Dual use research of concern
☒	☐	Plants

Methods

n/a	Involved in the study	
☒	☐	ChIP-seq
☒	☐	Flow cytometry
☒	☐	MRI-based neuroimaging

Antibodies

Antibodies used	The antibodies used in this study were: GluA1 (1:500; ab31232; Abcam), GluA2 (1:500; ab20673; Abcam), p-s845 (1:500; 36-8300; Invitrogen), p-s831 (1:500; AB5847; Millipore), LC3 (1:3000; NB100-2220; Novus Bio), p62 (1:500; H00008878-M01; Tebu-Bio), Synapsin 1/2 (1:1000; 106002; Synaptic System), SNAP-25 (1:1000; 111002; Synaptic System), Snapin (1:1000; 148002; Synaptic System), VAMP2 (1:1000; 104202; Synaptic System); Synaptophysin (1:1000, ab8049, Abcam), vGluT1 (1:1000; 135304; Synaptic

	System), CSPα (1:1000, AB1576, Millipore), PSD-95 (1:1000, 124011, Synaptic System), D1DR (1:1000, sc-33660, Santa Cruz), Tyrosine Hydroxylase (1:1000, AB152, Abcam), NMDAR1 (1:1000, 320500, Invitrogen), NMDAR2A (1:1000, M264, Sigma) and NMDAR2B (1:1000, 718600, Invitrogen). β-actin (1:5000, MAB1501, Millipore) was used as loading control.
Validation	We used antibodies already validated by previous studies (De Risi et al., 2020; Giordano et al., 2018) or by manufacturer for western blot or immunofluorescence. Moreover, control of specificity of immunolabeling were performed by omission of primary antibodies.

Animals and other research organisms

Policy information about studies involving animals; ARRIVE guidelines recommended for reporting animal research, and Sex and Gender in Research

Laboratory animals	Experiments were performed in CD1 outbred male mice, 9–10 weeks old at the beginning of experiments.
Wild animals	We did not use wild animals.
Reporting on sex	Experiments were performed in CD1 outbred male mice.
Field-collected samples	No field-collected samples were used in the study.
Ethics oversight	All procedures relating to animal care and treatments conformed to the guidelines and policies of the European Communities Council and were approved by the Italian Ministry of Health.

Note that full information on the approval of the study protocol must also be provided in the manuscript.
